# The cellular protein phosphatase 2A is a crucial host factor for Marburg virus transcription

**DOI:** 10.1128/jvi.01047-24

**Published:** 2024-08-28

**Authors:** Isabel von Creytz, Cornelius Rohde, Nadine Biedenkopf

**Affiliations:** 1 Institute of Virology, Philipps-University Marburg, Marburg, Germany; The Ohio State University, Columbus, Ohio, USA

**Keywords:** Marburg virus, filovirus, virus replication, protein phosphorylation, PP2A-B56, reverse genetics, LB-100 inhibitor

## Abstract

Little is known regarding the molecular mechanisms that highly pathogenic Marburg virus (MARV) utilizes to transcribe and replicate its genome. Previous studies assumed that dephosphorylation of the filoviral transcription factor VP30 supports transcription, while phosphorylated VP30 reduces transcription. Here, we focused on the role of the host protein phosphatase 2A (PP2A) for VP30 dephosphorylation and promotion of viral transcription. We could show that MARV NP interacts with the subunit B56 of PP2A, as previously shown for the Ebola virus, and that this interaction is important for MARV transcription activity. Inhibition of the interaction between PP2A and NP either by mutating the B56 binding motif encoded on NP, or the use of a PP2A inhibitor, induced VP30 hyperphosphorylation, and as a consequence a decrease of MARV transcription as well as viral growth. These results suggest that NP plays a key role in the dephosphorylation of VP30 by recruiting PP2A. Generation of recombinant (rec) MARV lacking the PP2A-B56 interaction motif on NP was not possible suggesting an essential role of PP2A-mediated VP30 dephosphorylation for the MARV replication cycle. Likewise, we were not able to generate recMARV containing VP30 phosphomimetic mutants indicating that dynamic cycles of VP30 de- and rephosphorylation are a prerequisite for an efficient viral life cycle. As the specific binding motifs of PP2A-B56 and VP30 within NP are highly conserved among the filoviral family, our data suggest a conserved mechanism for filovirus VP30 dephosphorylation by PP2A, revealing the host factor PP2A as a promising target for pan-filoviral therapies.

**IMPORTANCE:**

Our study elucidates the crucial role of host protein phosphatase 2A (PP2A) in Marburg virus (MARV) transcription. The regulatory subunit B56 of PP2A facilitates VP30 dephosphorylation, and hence transcription activation, via binding to NP. Our results, together with previous data, reveal a conserved mechanism of filovirus VP30 dephosphorylation by host factor PP2A at the NP interface and provide novel insights into potential pan-filovirus therapies.

## INTRODUCTION

Marburg virus (MARV) and the closely related Ebola virus (EBOV) belong to the family of *Filoviridae* within the order *Mononegavirales* (1, 2). Both viruses cause severe febrile illnesses in humans, which can lead to hemorrhages and multi-organ dysfunctions, and are associated with a lethality rate of up to 90% (3–6). Given the absence of licensed vaccines or therapeutics for MARV (7), the World Health Organization has classified MARV, like EBOV, as a priority pathogen since 2015 (8). The increasing frequency of MARV outbreaks in recent years in previously unaffected countries (2021 in Guinea, 2022 in Ghana, 2023 in Equatorial Guinea and Tanzania) emphasizes the high potential of these viruses to cause severe epidemics (5, 6), as seen before for EBOV in West Africa (2014–2016) with 28,610 infected people (11,308 deaths). Accordingly, it is imperative to enhance our understanding of the replication strategies of these viruses to identify potential targets for antiviral therapies.

Both MARV and EBOV contain a negative-sense RNA genome that encodes seven viral proteins: nucleoprotein NP, VP35, VP40, VP24, glycoprotein GP, transcription factor VP30, and the RNA-dependent RNA polymerase L (9–11). The viral genome has a length of 19 kb and is tightly encapsidated by NP, building together with the other viral nucleocapsid proteins L, VP35, VP30, and VP24 a helical nucleocapsid (12–15). While EBOV VP30 has been known for several years as an essential viral transcription factor that is indispensable for viral replication (16), the role of MARV VP30 in viral RNA synthesis was not clearly understood. Early reporter gene systems showed that MARV VP30 was not essential for viral transcription and replication (17) but enhances both (18, 19). However, data showing that siRNA knockdown of VP30 abrogates viral replication in combination with data demonstrating the failure to generate recombinant (rec) MARV without VP30 suggested an important role of VP30 in the tight regulation of MARV RNA synthesis (20, 21), comparable to EBOV VP30. Especially, recent data using MARV-specific multicistronic minigenomes (MG) suggest that VP30 regulates transcription reinitiation at the GP gene (22).

Similar to EBOV VP30 (23), MARV VP30 is a phosphoprotein, being phosphorylated at the N-terminal serine and threonine cluster ^40^SxSSxxSSxxSSxT^53^ (18, 24). Phosphorylation of MARV VP30 influences the interaction with NP (18, 24) as well as VP35 (18). Furthermore, recent data showed that dephosphorylation of MARV VP30 contributes to the regulation of viral RNA synthesis by enhancing viral transcription (18). Dephosphorylation of MARV VP30 could be inhibited by okadaic acid, a compound that blocks host cell protein phosphatases 1 (PP1) and 2A (PP2A) (25, 26), as well as by a PP1-specific inhibitor (18). Recently, we have elucidated the mechanism by which cellular PP2A dephosphorylates EBOV VP30 and thereby triggers viral transcription, with NP as a key factor for VP30 dephosphorylation (27). The regulatory subunit B56 of PP2A binds directly to EBOV NP via a specific binding motif (LxxIxE) in its C-terminal intrinsically disordered region (27). Simultaneous binding of VP30 to NP, mediated via a PPxPxY interaction motif (28, 29), places VP30 and PP2A in close spatial proximity. This facilitates the dephosphorylation of VP30 by PP2A at the NP interface and, hence, the activation of EBOV transcription (27). Interestingly, both binding motifs for PP2A-B56 and VP30 (LxxI/VxE and PPxPxY, respectively) are highly conserved among filoviral NPs. This suggests that a similar mechanism may be employed by other members of the *Filoviridae* family to regulate viral RNA synthesis (27). The interaction of a MARV NP peptide (containing the LxxVxE motif) with the B56 subunit of PP2A has already been demonstrated (27); however, the impact of this interaction on the MARV replication cycle, especially with respect to MARV VP30 dephosphorylation and transcription activation, was so far only speculative.

Here, we could demonstrate that the interaction between MARV NP and PP2A-B56 is important for MARV transcription. Inhibiting the PP2A-NP interaction via mutation of the LxxVxE binding motif, or using a PP2A inhibitor, resulted in reduced MARV transcription in life cycle model systems and induced hyperphosphorylation of VP30. A VP30 mutant mimicking permanent dephosphorylation of VP30 (VP30-A.A_6_.A) could rescue transcription activity in the presence of NP lacking interaction with PP2A-B56 (NP∆B56), suggesting general functionality of this mutant during viral transcription and replication and linking the transcription defect upon the missing interaction to VP30 hyperphosphorylation. The PP2A-specific inhibitor LB-100 significantly decreased viral titers and RNA levels in MARV infection in various relevant cell lines, emphasizing the importance of PP2A for efficient MARV RNA synthesis. Additionally, the generation of recMARV was not possible when the PP2A-B56 interaction site on NP was mutated (NP∆B56). The same was observed for VP30 mutants reflecting a permanent phosphorylation (VP30-D.D_6_.D) or dephosphorylation (VP30-A.A_6_.A), indicating that dynamic de- and rephosphorylation cycles of VP30 by essential host factors are indispensable for efficient replication of authentic MARV.

Altogether, our data indicate a crucial role of host factor PP2A for the MARV replication cycle by bringing a third player into the mechanistic play— NP—which acts as an essential scaffold protein recruiting both, PP2A and its substrate VP30, to induce VP30 dephosphorylation and thereby contributing to MARV transcription activation. Our findings emphasize a fundamental conserved mechanism utilized by different filoviruses (27), thus representing a promising candidate for the development of pan-filoviral therapeutic strategies.

## MATERIALS AND METHODS

### Cells

HuH7 [human hepatoma cells, fully matching the short tandem repeat (STR) reference profile of cell line HuH7], HEK293F (primary embryonic human kidney, fully matching the STR reference profile of cell line HEK293), and Vero C1008 (clone E6, Vero E6, ATCC CRL-1586, African green monkey kidney cells) cells were cultivated in Dulbecco’s modified Eagle medium (DMEM) supplemented with fetal bovine serum (10% for maintenance and 3% for experiments), penicillin (50 units/mL), streptomycin (50 mg/mL), and glutamine (2 mM) (10% resp. 3% DMEM ++) at 37°C and 5% CO_2_. THP-1 cells (DSMZ no.: ACC 16) were purchased from the Leibniz-Institut DSMZ GmbH. THP-1 cells were cultured in Roswell Park Memorial Institute 1640 medium (RPMI, Thermo Fisher Scientific) supplemented with fetal bovine serum (10% for maintenance and 3% for experiments), penicillin (50 units/mL), streptomycin (50 µg/mL), and glutamine (2 mM) (10% resp. 3% RPMI ++) at 37°C and 5% CO_2_. To differentiate THP-1 suspension cells into adherent macrophage-like cells, Phorbol 12-myristate 13-acetate (PMA, Sigma-Aldrich, P8139) was added. 3.3 × 10^4^ cells were seeded in 96-well plates, or 8 × 10^4^ cells were seeded in 24-well plates (Corning Primaria, Waltham, MA, USA) and stimulated with 200 nM PMA. After 48 h, the medium was replaced with a fresh one, and the cells were cultured for further 5 days until the infections were started.

### Viruses

All MARV experiments were performed at the BSL-4 facility of the Philipps-University Marburg according to national regulations. Infections were performed as described in von Creytz *et al.* (30). Briefly, a MARV strain Musoke (GenBank accession number DQ217792) stock was produced in Vero E6 cells infected with a multiplicity of infection (MOI) of 0.1 and afterward incubated for 7 days at 37°C. Viral titers were calculated based on an immunoplaque assay (PFU/mL). For this, confluent Vero E6 cells were infected in a 24-well plate with different dilutions of the virus. The inoculum was replaced by 500 µL 2% carboxymethylcellulose in Minimum Essential Medium [MEM containing penicillin (50 units/mL) and streptomycin (50 mg/mL)], and the cells were incubated at 37°C for 4 days. Afterward, the cells were fixed with 4% paraformaldehyde (PFA)/DMEM for 48 h, discharged from the BSL-4 laboratory and prepared as well as permeabilized for intracellular immunofluorescence staining. Immunofluorescence staining was performed with a goat α-MARV serum (dilution 1:2,000) (31) and a donkey α-goat IgG (H + L) Cross-Adsorbed Alexa Fluor 488 secondary antibody (dilution 1:400). Plaques were counted using a fluorescence microscope. The number of plaques was multiplied by the dilution factor to calculate the titer, which was expressed as PFU/mL.

### Cloning of MARV plasmids

Plasmids encoding wildtype (wt) or HA-tagged viral proteins of MARV Musoke as well as a monocistronic (1cis) MARV-specific MG, a firefly luciferase and the T7 polymerase were described elsewhere (17, 19, 32). The cloning of the pCAGGS HA-VP30 mutants mimicking permanent de-/phosphorylation (VP30-A.A_6_.A: S40A, S42A, S43A, S46A, S47A, S50A, S51A, T53A; VP30-D.D_6_.D: S40D, S42D, S43D, S46D, S47D, S50D, S51D, T53D) and the pCAGGS NPΔB56 mutant (L525A, V528A) were performed using a multisite-directed mutagenesis kit (QuikChange Multi Site-Directed Mutagenesis Kit, Agilent) according to the manufacturer’s recommendations. Plasmids encoding YFP-LxxIxE and YFP-AxxAxA (both encoding the Venus version of YFP, vector pCDNA-FRT-TO) and YFP-B56α (vector pCDNA-FRT-TO) were described elsewhere (27).

Cloning of a tetracistronic (4cis) MARV (strain Musoke) MG (pAndy-MARV-4cis) that encodes, in addition to a *Renilla* luciferase reporter gene, the viral proteins VP40, GP, and VP24 was performed using Gibson assembly (Gibson Assembly Master Mix, NEB) according to the manufacturer’s protocol on the basis of the previously generated 1cis MARV-specific MG (17). The following inserts were used: MARV 3′ leader sequence, *Renilla* luciferase gene, non-coding region (NCR) between NP and VP35, VP40 gene, NCR between VP40 and GP, GP gene, NCR between VP30 and VP24, VP24 gene, 5′ trailer sequence.

Generation of a full-length (FL) plasmid encoding the whole MARV genome is based on a three-part cassette system as described elsewhere (30, 33). Respective cassette plasmids encoding either NP, or VP30, were used for the introduction of mutations mimicking permanent de-/phosphorylation (VP30-A.A_6_.A, VP30-D.D_6_.D) and/or mutations within the LxxVxE-binding motif within NP (NPΔB56) using multisite-directed mutagenesis kit (Agilent) according to the manufacturer’s recommendations.

All plasmids were verified by Sanger sequencing by Microsynth Seqlab. Detailed cloning strategies and primer sequences are available on request.

### MARV-specific MG assay

**Fig 1:**
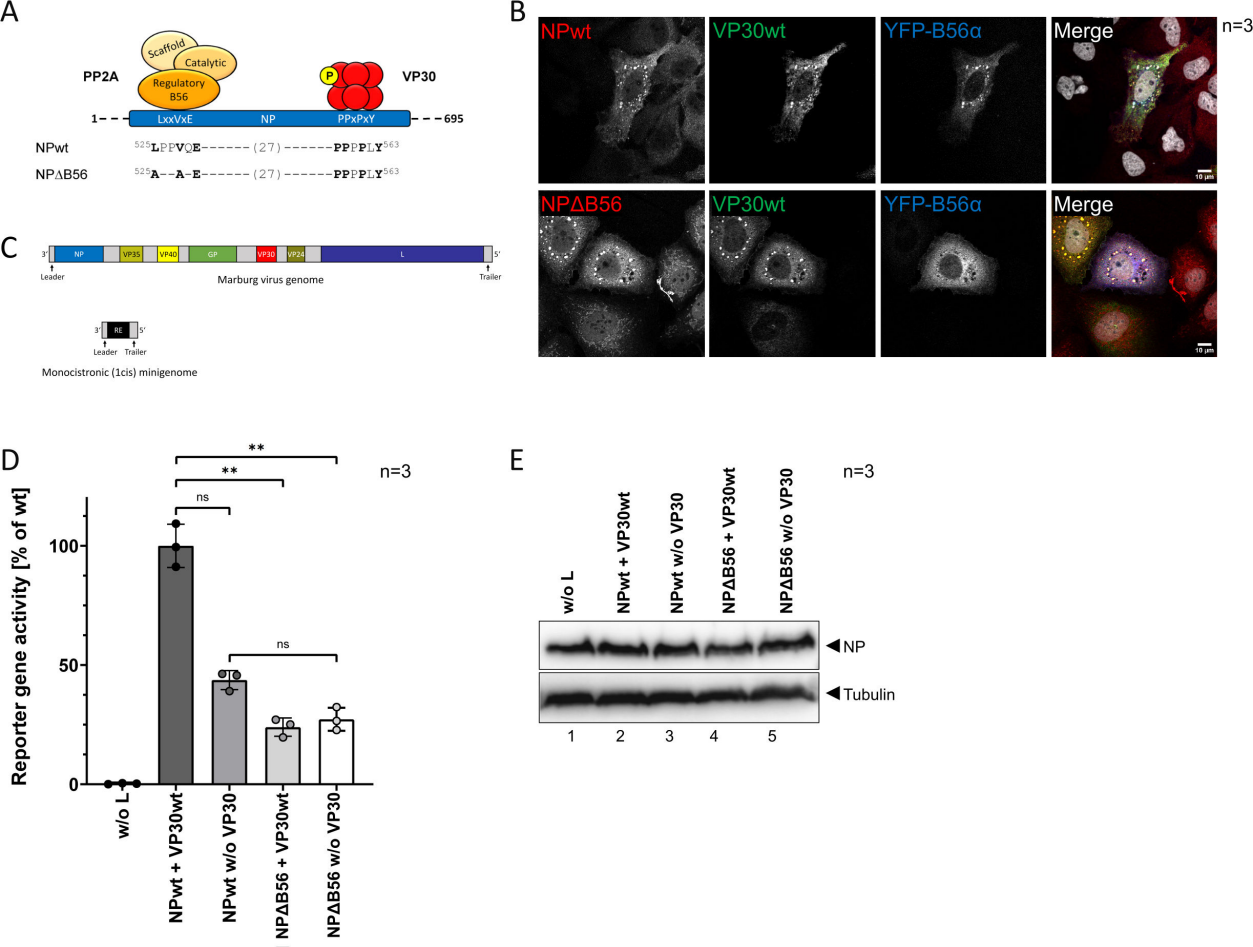
(**A**) Schematic representation of the binding motifs of the PP2A regulatory subunit B56, and VP30 in NP. Point mutations in LxxVxE-binding motif of NPΔB56 mutant preventing interaction of NP and PP2A-B56 are indicated. (**B**) Inclusion body formation of NP and NPΔB56. HuH7 cells were transfected with plasmids encoding VP30wt, YFP-B56, and NPwt, or NPΔB56. Cells were fixed 24 hpt with 4% PFA, and immunofluorescence analysis was performed using NP- (red) and VP30- (green) specific antibodies. YFP-signal (blue) was intensified using a GFP booster. Nuclei were stained with DAPI (gray). Scale bar: 10 µM. Three independent experiments were performed. (**C**) Schematic representation of the used monicistronic (1cis) MARV-specific MG containing a *Renilla* reporter gene in comparison with the FL genome. (**D**) MARV-specific MG assay. HEK293F cells were transfected with plasmids encoding NPwt, or NPΔB56, and L, VP35, VP30, a 1cis MG, as well as a T7 polymerase and a firefly luciferase for normalization. A negative control without L (w/o L) and a negative control without VP30 (w/o VP30) were included. Cells were harvested for luciferase measurement 24 hpt; standard deviation (SD) is indicated by error bars, and stars indicate statistical significance (ns, not significant; **P-value ≤ 0.01). Three independent experiments were performed. (**E**) WB analysis of cell lysates from C using monoclonal antibodies specific for MARV NP and for Tubulin.

**Fig 2:**
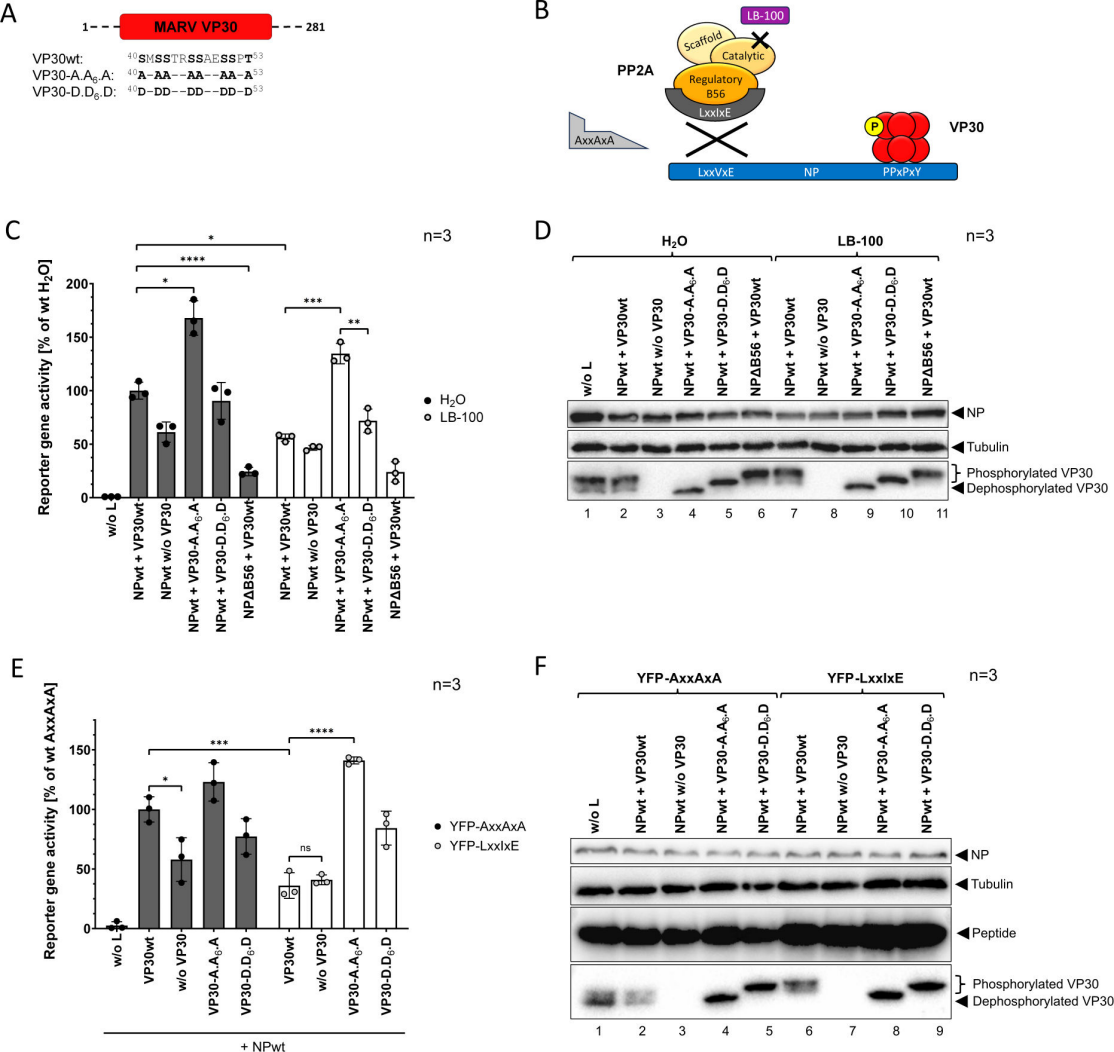
(**A**) Scheme of the N-terminal VP30 phosphorylation sites and the mutations to generate a VP30-A.A_6_.A mutant mimicking permanent dephosphorylation and a VP30-D.D_6_.D mutant mimicking permanent phosphorylation. (**B**) Inhibition of PP2A either by the small-molecule inhibitor LB-100 or a peptide inhibitor specific to the PP2A subunit B56 (YFP-LxxIxE; control peptide YFP-AxxAxA). (**C**) MG assay in the presence of the PP2A inhibitor LB-100. HEK293F cells were transfected with plasmids encoding NPwt, or NPΔB56, and L, VP35, VP30wt, VP30-A.A_6_.A, or VP30-D.D_6_.D, a monocistronic (1cis) MG encoding a *Renilla* luciferase, as well as a T7 polymerase and a firefly luciferase for normalization. Negative controls without L (w/o L) and without VP30 (w/o VP30) were included. Cells were treated with a medium containing either H_2_O, or 2.5 µM LB-100 starting at 4 hpt. Cells were harvested for luciferase measurement 24 hpt; SD is indicated by error bars, and stars indicate statistical significance (*P-value ≤ 0.05; **P-value ≤ 0.01, ***P-value ≤ 0.001, ****P-value ≤ 0.0001). Three independent experiments were performed. (**D**) WB analysis of the 1cis MG assays shown in C using antibodies specific for MARV NP, or VP30, and for Tubulin. (**E**) MARV-specific MG assay in the presence of the peptide inhibitor YFP-LxxIxE. HEK293F cells were transfected with plasmids encoding YFP-LxxIxE, or YFP-AxxAxA, and NPwt, L, VP35, VP30wt, VP30-A.A_6_.A, or VP30-D.D_6_.D, a 1cis MG, as well as a T7 polymerase and a firefly luciferase for normalization. Negative controls without L (w/o L) and without VP30 (w/o VP30) were included. Cells were harvested for luciferase measurement 24 hpt; SD is indicated by error bars, and stars indicate statistical significance (ns, not significant; *P-value ≤ 0.05; ***P-value ≤ 0.001, ****P-value ≤ 0.0001). Three independent experiments were performed. (**F**) WB analysis of the MG assays shown in E using antibodies specific for MARV NP, or VP30, for Tubulin, and YFP-tag.

MARV-specific 1cis MG assays were performed in HEK293F cells (8 × 10^5^ cells/6 well). Cells were transfected with plasmids encoding the viral nucleocapsid proteins essential for viral transcription and replication (L, NP, VP35, VP30) together with a T7-driven 1cis MARV-specific MG encoding a *Renilla* luciferase (17, 19). Plasmids were transfected using the following DNA concentrations: 500 ng NP, 100 ng VP35, 100 ng VP30, 1,500 ng L, 500 ng 1cis MG, and 100 ng T7-polymerase. In addition, a plasmid encoding a firefly luciferase (25 ng, pGL4 vector) was additionally transfected for normalization. For the MG assays with the PP2A-B56-specific peptide inhibitor, an additional 250 ng of either the control peptide YFP-AxxAxA or the inhibitor peptide YFP-LxxIxE was transfected. TransIT-LT-1 (Mirus Bio LLC) was used for transfection according to the manufacturer’s protocol. For the MG assays with the PP2A inhibitor LB-100, 2.5 µM LB-100 (Selleck)/H_2_O, or H_2_O (0.25%) as control were added to the medium 4 h after transfection (3% DMEM ++), the cells were incubated at 37°C. Cells were washed 24 hours post transfection (hpt) once with phosphate-buffered saline (PBS_def_, PBS deficient of MgCl_2_ and CaCl_2_), harvested in fresh PBS_def_, and lysed in 100 µL 1× Passive Lysis buffer (Promega) at room temperature (RT) for 15 min. 5 µL of cell lysate each was used to measure *Renilla* luciferase (MARV-specific reporter gene activity) or firefly luciferase activity with *Renilla*-Juice or Beetle-Juice BIG KITs (bot PJK), respectively, in a CentroLB 960 luminometer (Berthold Technologies). *Renilla* luciferase raw data were normalized with the corresponding firefly luciferase raw data; subsequently, all normalized *Renilla* data were normalized to the positive control (NPwt + VP30wt). 30 µL of cell lysate was mixed with sodium dodecyl sulfate (SDS) sample buffer (25% glycerol, 2.5% SDS, 125 mM Tris pH 6.8, 125 mM dithiothreitol, 0.25% bromophenol blue) and boiled for 10 min at 95°C for Western blot (WB) analysis. One-way (Fig. 1D) or two-way (Fig. 2C and E) ANOVA with Tukey’s multiple comparison test was performed with the logarithm of the presented data for statistics using GraphPad Prism (version 10.0.2).

### Transcription- and replication-competent virus-like particle assay

1cis or 4cis transcription- and replication-competent virus-like particle (trVLP) assays were performed in HuH7 cells (2 × 10^5^ cells/6 wells). For passage 0 (P0), all plasmids were transfected as described for the 1cis MG except that 2.5 µg of the 4cis MG was transfected. In the case of the 1cis trVLP assay, additional plasmids of GP (500 ng), VP40 (500 ng), and VP24 (70 ng) were transfected, as already described elsewhere (19). After transfection, cells were incubated for 72 h at 37°C. The cell lysates were harvested, luciferase signals were measured, and samples for WB analysis were taken as described for 1cis MG assays. Generated trVLPs were purified from the supernatant of the cells by centrifugation for 10 min at 2,500 rpm to spin down cell residues. Second, the supernatants were centrifugated via ultracentrifugation (40,000 rpm for 2 h at 4°C). The pellets containing trVLPs were resuspended in 100 µL sterile PBS_def_, of which 80 µL was mixed with 320 µL 0% DMEM ++ and used as inoculum to infect naïve passage 1 (P1) cells (HuH7, 1 × 10^5^ cells/12 well). P1 cells were incubated with the inoculum for 2 h at 37°C, and the plate was carefully swiveled every 15 min. Afterward, 1.6 mL 3% DMEM ++ was added, and cells were incubated for 72 h at 37°C. Thereafter, the P1 cells were washed once with PBS_def_, harvested in fresh PBS_def_, and lysed in 50 µL 1× Passive Lysis buffer (Promega) at RT for 15 min. 10 µL cell lysate was used to measure *Renilla* luciferase signals as described for MG assays. Residual 20 µL of the ultracentrifugated 1cis trVLPs was split into two different samples for WB analysis: 10 µL as untreated trVLP control, 10 µL trVLPs were digested with 1 µL Proteinase K (AJ Innuscreen, 1 mg/mL stock solution) for 1 h at 37°C. Afterward, Proteinase K activity was stopped with 1 µL phenylmethylsulfonylfluorid (100 mM stock solution, Merck) for 10 min at RT.

One-way ANOVA with Tukey’s multiple comparison test was performed with the logarithm of the presented data for statistics using GraphPad Prism (version 10.0.2).

### Strand-specific quantitative RT-PCR

The transfection of P0 HuH7 cells with the 4cis trVLP assay setting was performed as described above. Cells were washed at 72 hpt with PBS_def_ and resuspended in 1 mL fresh PBS_def_. The cells were spun down for 5 min at 8,000 rpm and 4°C. Afterward, RNA extraction using the RNeasy Mini Kit (Qiagen) was performed according to the manufacturer’s protocol. In addition to the DNase digest on the column included in the kit protocol, a second DNase digest in solution was performed using Ambion DNase I (Thermofisher) according to the manufacturer’s protocol. Thereafter, the RNA was purified again with the RNeasy Mini Kit (Qiagen) according to the manufacturer’s protocol. Those steps were necessary to eliminate residual transfected MG plasmid. For strand-specific cDNA synthesis, primer detecting negative strands of the *Renilla* gene (27), positive strands of the *Renilla* gene (27), and positive strands of the trailer region (5′-AAGCTCAAGGATTCACCCAATGT-3′) were used to distinguish genomic RNA, antigenomic RNA, and mRNA. Using 5 µL RNA, cDNA synthesis was conducted with RevertAid First strand cDNA synthesis kit (Thermofisher) according to the manufacturer’s protocol. Quantitative real-time PCR (qRT-PCR) was performed with a StepOne Real-Time PCR system (Applied Biosystems) using the Platinum Taq DNA Polymerase High Fidelity (Thermo Fisher Scientific Invitrogen) using 5 µL cDNA and 12.5 µmol MgSO_4_. In addition, a probe specific for the *Renilla* luciferase (5′-CCACATATTGAGCCAGTAGCGCGG-3′) containing 5′ FAM (6-FAM Phosphoramidit) and 3′ DDQ-1 (Deep Dark Quencher-1, Biomers) was used for qRT-PCR analysis (27). As standard, serial 10-fold dilutions (10^3^–10^7^) of the 4cis MG plasmid were used to calculate the amount of RNA copies in the samples. Following PCR conditions were used: initial denaturation of cDNA was performed at 95°C for 2 min, followed by 40 cycles with denaturation at 95°C for 15 s, annealing at 60°C for 30 s, and elongation at 95°C for 15 s. The qRT-PCR procedure was already published elsewhere (27). Measured RNA copy levels were normalized to the positive control (NPwt + VP30 wt). One-way ANOVA with Tukey’s multiple comparison test was performed with the logarithm of the presented data (for each RNA species separately) for statistics using GraphPad Prism (version 10.0.2). Only statistical differences are shown in the figure.

### Rescue of recMARV mutants

Rescues were performed as described elsewhere (30, 33). Briefly, a mixed culture of HuH7 cells and Vero E6 cells (each cell line: 1 × 10^5^ cells/6 well, P0) was transfected using plasmids encoding the respective mutated FL MARV genome (2 µg), T7 polymerase (0.5 µg), and helper plasmids coding for L (2 µg), NP (0.5 µg), VP35 (0.1 µg), and VP30 (0.1 µg). As a positive control, the FL plasmid of MARV Musoke wt was used. Transfections were performed in technical duplicates using Trans-IT-LT-1 (Mirus Bio LLC) according to the manufacturer’s protocol. A medium change was performed 4 hpt to remove residual transfection reagent. Supernatants were transferred 7 days post transfection onto fresh Vero E6 cells (P1). The rescue was continued until P3. The development of cytopathic effect (CPE) was monitored, and the supernatants were analyzed for the presence of VP40 by WB analyses, as well as for viral RNA. Viral RNA was extracted using the QIAamp Viral RNA Mini Kit (Qiagen) according to the manufacturer’s protocol and subsequently transcribed by Transcriptor One-Step RT-PCR Kit (Roche) using MARV-specific primers covering mutated regions. The resulting cDNA was sequenced by Microsynth Seqlab (Sanger sequencing).

### Indirect immunofluorescence analysis

HuH7 cells (2 × 10^5^ cells/6 well, seeded on cover slips) were transfected with plasmids encoding viral proteins VP30 (300 ng), and NPwt or NPΔB56 (300 ng), as well as a plasmid encoding YFP-B56α (300 ng) (Fig. 1D). For the second immunofluorescence analysis, HuH7 cells (2 × 10^5^ cells/6 well, seeded on cover slips) were transfected with plasmids for a 1cis trVLP assay, as described above (Fig. 6A). TransIT-LT-1 (Mirus Bio LLC) was used for transfection according to the manufacturer’s protocol. For both experiments, 24 hpt (first experiment, Fig. 1D) or 72 hpt (second experiment, Fig. 6A) cells were fixed with 4% PFA/DMEM for 20 min. After washing with PBS_def_, cells were permeabilized with 0.1% Triton X-100 in PBS_def_ for 10 min, treated with 0.1 M glycine for 10 min, and afterward incubated in blocking buffer (containing 2% bovine serum albumin; 5% Glycerin; 0.2% Tween 20; 0.05% NaN_3_ in PBS_def_) for 10 min. Primary antibodies (monoclonal mouse α-VP30 clone 11-6-11, dilution 1:50; polyclonal chicken α-NP 102, dilution 1:100) were diluted in blocking buffer, staining occurred for 1 h at RT. Cover glasses were washed three times in fresh PBS_def_. Finally, staining with fluorescence-labeled secondary antibodies [goat α-chicken IgY (H + L) AlexaFluor 594, dilution 1:400, Invitrogen; goat α-mouse IgG (H + L) Cross-Adsorbed AlexaFluor 488, dilution 1:400, Invitrogen] was followed. To increase the YFP signal in the first experiment (Fig. 1D), a GFP-booster Atto488 was used (Chromotek, dilution 1:500). DAPI (4′,6-diamidino-2′-phenylindol) was added at a dilution of 1:2,000. Two wash steps in PBS_def_ and one wash step in ddH_2_O each 5 min at RT were followed. The cover glasses were analyzed using the Leica TCS SP5 II confocal laser scanning microscope and a HyD3 detector.

### Quantification of immunofluorescence data

Unprocessed confocal laser scanning microscope-generated pictures were analyzed for overlapping signals using ImageJ/Fiji v.1.52i. Original layers of red (NP) and green (VP30) channels were compared using a pixel-based analysis and summarized in a Pearson’s correlation coefficient (PCC) using the Coloc2 tool with Coste’s threshold regression. Data were analyzed for normal distribution and variance. This quantification procedure was already published elsewhere (34). Kruskal-Wallis tests were performed for statistics of PCC data using GraphPad Prism (version 10.0.2).

### WB analysis

Proteins were separated using 10% or 12% SDS polyacrylamide gels and transferred onto polyvinylidene difluoride membranes. Membranes were blocked in 10% non-fat dry milk in PBS_def_ for 1 h at RT. Staining with the monoclonal primary antibodies (mouse α-GP clone 50-6-10, dilution 1:500; mouse α-NP clone 59-9-10, dilution 1:2,000 or 1:500 for 1cis trVLPs; mouse α-VP40 clone 40-2-2, dilution 1:1,000 or 1:500 for 1cis trVLPs; mouse α-VP30 clone 11-6-11, dilution 1:1,000 or 1:500 for 1cis trVLPs; all Institute of Virology), that are specific to MARV Musoke, as well as a polyclonal Venus-specific (YFP-tag) antibody for staining of the peptides (goat α-Venus, MyBioSource, dilution 1:4,000), or Tubulin [mouse α-Tubulin, Sigma-Aldrich (#T9026, dilution 1:4,000)], was performed in PBS_def_ supplemented with 1% non-fat dry milk and 0.1% Tween 20 overnight at 4°C or for 1 h at RT. WB detection was performed with peroxidase-conjugated secondary antibodies (goat α-mouse HRP, 1:40,000, Dako; donkey α-goat POD, 1:40,000, Dianova) using the SuperSignal West Femto Maximum Substrate (Thermo Scientific) or Luminata Forte Western HRP Substrate (Merck) and ChemiDoc (BioRad).

### Cell viability assay

HuH7 cells (1 × 10^4^ cells/96 well) and HEK293F cells (2 × 10^4^ cells/96 well) were incubated in 3% DMEM ++ containing 0.25 µM, 0.5 µM, 1 µM, 2.5 µM, 5 µM, 7.5 µM, 10 µM, or 100 µM LB-100 (Selleck) H_2_O (1 mM LB-100 stock solution) or H_2_O (10%) as control for 24 h at 37°C. About half of the seeded (3.3 × 10^4^ cells/96 well) monocyte-like THP-1 cells differentiated into macrophage-like cells (~1.65×10^4^ cells/96 well), which were subsequently incubated in 3% RPMI medium containing the same concentrations of LB-100 as described for HuH7 cells and HEK293F cells. For Vero E6 cells (1.5 × 10^4^ cells/96 well), LB-100 concentrations of 5 µM, 50 µM, 75 µM, 100 µM, and 250 µM (10 mM LB-100 stock solution), or H_2_O (2.5%) as control were used in 3% DMEM. In addition, control wells containing only medium and no cells were used as a negative control to measure plate backgrounds. For each sample, eight technical replicates were performed within each of the three biological replicates. After 24 h, measurement of the cellular viability was conducted using CellTiter-Glo^©^ 2.0 reagent (Promega) according to the manufacturer’s protocol. One-way ANOVA with Tukey’s multiple comparison test was performed with the logarithm of the presented data for statistics using GraphPad Prism (version 10.0.2).

### MARV infection with inhibitor LB-100

HuH7 (1 × 10^5^ cells/12 well) or VeroE6 cells (1.5 × 10^5^ cells/12 well) were infected with MARV using MOIs of 0.01 or 0.1. After the removal of the virus-containing inoculum, cells were washed once in PBS_def_ and were further incubated for 24 h at 37°C in 3% DMEM ++ containing either LB-100 (Selleck)/H_2_O (HuH7 cells: 0.5 µM, 1 µM, 2.5 µM, 5 µM using a 1 mM LB-100 stock solution; Vero E6: 5 µM, 50 µM, 100 µM using a 1 mM LB-100 stock solution) or H_2_O (HuH7 cells: 0.5%, Vero E6: 10%) as control. THP-1 cells (~4 × 10^4^ cells/24 well, corresponding to 50% of the originally seeded cells differentiated into macrophage-like cells) were infected with MARV using MOI 0.1 or 1 and cultivated in 3% RPMI medium containing LB-100 (1 µM, 2.5 µM, 5 µM, 7.5 µM using a 1 mM LB-100 stock solution) or H_2_O (0.75%). After 24 h, cells were harvested in RLT buffer from the RNeasy Mini Kit (Qiagen), and viral RNA copies in the cell lysates were examined via qRT-PCRs (described below). In addition, supernatants were sampled at 24 hpi, and viral titers in the supernatants were determined by 50% tissue culture infectious dose (TCID_50_) assays using Vero E6 cells. Titers were calculated as TCID_50_/mL with the Spearman-Kärber method (35).

For qRT-PCRs, cell lysates harvested at 24 hpi in RLT buffer were used for RNA extraction via the RNeasy Mini Kit (Qiagen) according to the manufacturer’s protocol. Subsequently, cDNA synthesis was performed with 300 ng RNA each using LunaScript RT SuperMix (NEB) according to the manufacturer’s protocol. The qRT-PCR was performed with a qTOWER PCR system (Roth) using the Luna Universal qPCR Master Mix (NEB) according to the manufacturer’s protocol with 0.9 µL cDNA, as well as MARV VP40 (strain Musoke) specific primers (5′-ATCTGCATATAACGAGCGAAC-3′ and 5′-CTTCACGCAACATTCTGAGT-3′), and cellular 18S ribosomal RNA primers (HuH7 cells and THP-1 cells) for normalization (5′-GCGGCGGAAAATAGCCTTTG-3′ and 5′-GATCACACGTTCCACCTCATC-3′), or cellular Tubulin primers for Vero E6 cells (5′-GGCCGTGTTTGTAGACTTGG-3′ and 5′-CTTCCTTGCCTGTGATGAGC-3′). Following PCR conditions were used: initial denaturation of cDNA was performed at 95°C for 1 min, followed by 45 cycles with denaturation at 95°C for 15 s, annealing at 60°C for 30 s. A melting curve was measured starting at 60°C and increasing in steps of 0.3°C/15 s to 95°C. The analysis was carried out via 2^−ΔΔ*Ct*^ normalized to the positive control only treated with H_2_O and cellular 18S ribosomal RNA/Tubulin RNA levels to exclude residual cytotoxic effects. One-way ANOVA with Tukey’s multiple comparison test was performed with the logarithm of the presented data (for each MOI separately) for statistics using GraphPad Prism (version 10.0.2).

## RESULTS

### Interaction of NP with the cellular phosphatase PP2A is important for MARV transcription

To investigate the role of the host factor PP2A during MARV replication, particularly concerning MARV VP30 dephosphorylation, we generated a NP mutant lacking the interaction motif for PP2A-B56 via two point mutations in the previously described interaction motif LxxVxE (NPΔB56, Fig. 1A) (27). Since NP drives the formation of inclusion bodies, which are a hallmark of infection and sites of RNA synthesis (36, 37), we initially examined whether the mutations affected inclusion body formation and localization of an additionally transfected B56 subunit of the PP2A complex and VP30 (Fig. 1B). Wildtype NP (NPwt) recruits both VP30 and the YFP-tagged B56 into NP-induced inclusion bodies. We did not observe any difference in inclusion body formation by NPΔB56, compared to NPwt. As the expression of both proteins was not synchronized during transfection, we found typical NP-induced inclusions ranging from small (representing earlier time points) to larger inclusions (representing later time points) for both, NPwt and NPΔB56 (38). This indicates that the ability of NPΔB56 to form inclusion bodies was still preserved after introducing the mutations. The distribution of VP30, which is recruited into NP inclusions, was unaffected in the presence of NPΔB56 suggesting a functional interaction. In contrast, B56 was not enriched in NPΔB56-induced inclusion bodies (Fig. 1B). These findings confirmed, in addition to previous data with a MARV NP peptide (27), a missing PP2A-B56 interaction upon mutation of the B56 motif also for FL NP.

We next assessed the NPΔB56 activity in monocistronic (1cis) MARV-specific MG reporter assays. This model system enables the investigation of viral transcription of highly pathogenic viruses on BSL-1/2 level by using a reporter gene (*Renilla* luciferase) that is flanked by regulatory viral 3′ leader and 5′ trailer sequences as MARV-specific MG (Fig. 1C) (16, 17, 19). The T7-transcribed MARV-specific MG RNA is recognized, replicated, and transcribed by the viral nucleocapsid complex consisting of the viral proteins L, NP, VP35, and VP30 (17). *Renilla* luciferase activity of the MG is measured 48 hpt and reflects MARV-specific transcription. Negative controls were represented by omitting the viral polymerase L or omitting VP30. While omitting L leads to a complete inhibition of MARV RNA synthesis, omitting VP30 reduces viral transcription to 50% (Fig. 1D) similar to previously published results (18). When comparing NPΔB56 with NPwt, transcription activity was significantly reduced indicating that the interaction between NP and PP2A is important for viral transcription. Interestingly, transcription activity without VP30 (NPΔB56 w/o VP30) was slightly decreased compared to NPwt (NPwt w/o VP30), suggesting an additive inhibitory effect of NPΔB56 independent of VP30 dephosphorylation, albeit this was not statistically significant (Fig. 1D). WB analysis confirmed similar expression levels of NPΔB56 compared to NPwt (Fig. 1E).

Taken together, these data suggest that the interaction of cellular PP2A via its subunit B56 with MARV NP is important for MARV transcription.

### PP2A-B56 dephosphorylates MARV VP30 thereby promoting viral transcription

Since the interaction of EBOV NP with PP2A directly influenced the phosphorylation status of EBOV VP30 (27), we were interested in whether the same holds true for the respective MARV proteins. To link the attenuated transcriptional activity upon prevented PP2A-B56 recruitment (NPΔB56) to an altered dephosphorylation level of VP30, we included different phosphomimetic VP30 mutants in the MG assay setting as further controls (Fig. 2A). Phosphorylatable serine and threonine residues within the N-terminal VP30 serine/threonine cluster (^40^SxSSxxSSxxSSxT^53^) known to be important for MARV transcription (18, 24) were either mutated to alanine to simulate permanent dephosphorylation (VP30-A.A_6_.A) or to aspartic acid to imitate a permanent phosphorylation at these sites (VP30-D.D_6_.D). We introduced phosphomimetic mutations within the whole cluster to observe the clearest possible effects on viral transcription in contrast to previous studies that focused on single residues (18, 24). Dephosphorylation-mimicking VP30-A.A_6_.A exhibited an enhanced promotion of MARV transcription in MG assays when compared to VP30wt (Fig. 2C, gray bars) highlighting the positive impact of VP30 dephosphorylation on viral transcription (18). Conversely, the VP30-D.D_6_.D mutant demonstrated a slight reduction in reporter gene activity toward levels without VP30, albeit not as strong as NPΔB56 (Fig. 2C). In parallel, we treated the cells with the inhibitor LB-100 targeting the catalytic subunit of PP2A (Fig. 2B). LB-100 is a well-characterized competitive small-molecule inhibitor against PP2A that has previously undergone testing in clinical trials for its anti-cancer properties (39). Dose-dependent cytotoxic effects of LB-100 in HEK293F cells were tested before, showing onset of cytotoxicity at concentrations of 5 µM and above (Fig. S1A). For this reason, we treated cells with a concentration of 2.5 µM LB-100 in MG assays. Interestingly, LB-100 significantly reduced viral transcription in the presence of NPwt and VP30wt (Fig. 2C, white vs gray bars). In contrast, treatment with LB-100 had no effect on viral transcription in the presence of both phosphomimetic VP30 mutants (VP30-A.A_6_.A and VP30-D.D_6_.D) nor in case of NPΔB56 when compared with water-treated controls (Fig. 2C, white vs gray bars). This highlights again the importance of PP2A for the activation of MARV transcription and further suggests that the negative effect of PP2A inhibition on viral transcription is linked to VP30 hyperphosphorylation.

To test this hypothesis, we conducted a WB analysis of the MG assay samples. MARV VP30wt can be detected as a distinguishable double band (Fig. 2D) (24), whereby the upper band corresponds to phosphorylated VP30 and the lower band to dephosphorylated VP30 (24). This could be demonstrated by both phosphomimetic VP30 mutants, each detectable as a single band. The dephosphorylation-mimicking VP30-A.A_6_.A was detected notably below (Fig. 2D, lane 4) the phosphorylation-mimicking VP30-D.D_6_.D mutant (Fig. 2D, lane 5). In the presence of NPΔB56 (Fig. 2D, lane 6), the majority of VP30 is shifted even above the upper band suggesting hyperphosphorylation of VP30 when the interaction of PP2A-B56 with NP is prevented. Inhibition of PP2A by LB-100 inhibitor treatment likewise revealed hyperphosphorylation of VP30wt (Fig. 2D, lane 7). When compared to water-treated cells, VP30wt shows a clearly upward-shifted band (Fig. 2D lane 2 vs lane 7) similar to VP30wt in the presence of NPΔB56 (Fig. 2D, lane 6 and lane 11). Treatment with LB-100 had no effect on the phosphorylation state of both VP30 phosphomimetic mutants, VP30-A.A_6_.A and VP30-D.D_6_.D, respectively (Fig. 2D, lane 9 and 10), emphasizing the hyperphosphorylation-induced shift of VP30wt upon LB-100 treatment.

Since LB-100 blocks phosphatase activity of PP2A, we wanted to assess whether a specific inhibition of the interaction of the B56 subunit with NP would have a similar impact on transcription activation and VP30 dephosphorylation. For this, we transfected plasmids encoding a competitive peptide inhibitor carrying the LxxIxE motif (YFP-LxxIxE, Fig. 2B) or, as a negative control, an AxxAxA motif (YFP-AxxAxA) (27). Overexpression of the specific competitive peptide inhibitor results in the interception of the PP2A subunit B56 and thereby prevents its binding to NP. The phosphatase activity of PP2A via other regulatory subunits remains unaffected as well as the NP-binding site (Fig. 2B). Transfection of the control peptide YFP-AxxAxA had no effect on transcription levels (Fig. 2E, gray bars). In contrast, the PP2A-B56-specific peptide inhibitor YFP-LxxIxE led to a significant reduction of reporter gene activity in the case of VP30wt (Fig. 2E, white bars) to comparable levels as omitting VP30 (Fig. 2E, gray and white bars). Again, viral transcription was unaffected in the presence of the dephosphorylation-mimicking VP30-A.A_6_.A suggesting a phosphorylation-specific effect on transcription in the case of VP30wt due to the competitive peptide inhibitor. WB analysis revealed no effect on the respective VP30 phosphorylation status in the presence of the control peptide YFP-AxxAxA (Fig. 2F, lanes 2–5). However, VP30wt phosphorylation changed in the presence of the specific inhibitor YFP-LxxIxE (Fig. 2F). Here, the prevention of PP2A-B56 binding to NP by the specific peptide inhibitor led to VP30wt hyperphosphorylation (Fig. 2F, lane 2 vs lane 6) similar to treatment with LB-100 (Fig. 2D, lane 7) or in the presence of NPΔB56 (Fig. 2D, lane 6). The phosphomimetic VP30 mutants VP30-A.A_6_.A and VP30-D.D_6_.D were still unaffected by the specific peptide inhibitor (Fig. 2F, lanes 8 and 9).

In summary, these results highlight the importance of PP2A-B56 in facilitating VP30 dephosphorylation, thereby promoting efficient MARV transcription.

### MARV primary transcription depends on PP2A-B56 interaction with NP

**Fig 3:**
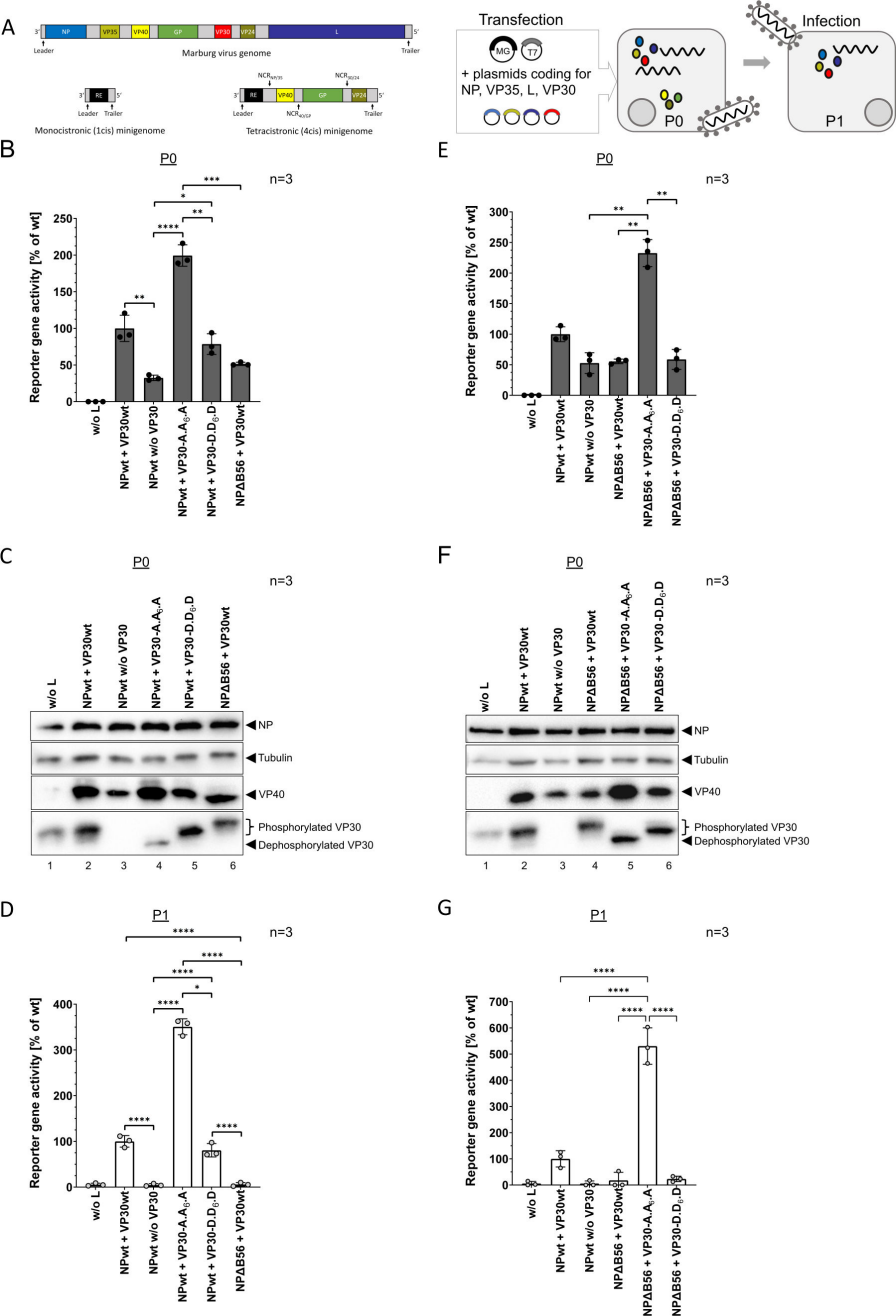
(**A**) Schematic representation of the generated tetracistronic (4cis) MARV-specific MG in a trVLP assay. Cells (**P0**) were transfected with plasmids encoding the viral nucleocapsid proteins NP, L, VP35, and VP30 together with the 4cis MG encoding a *Renilla* luciferase as a reporter gene as well as a T7 polymerase and a firefly luciferase. After transcription and replication of the 4cis MG, nucleocapsids assemble and are transported to the plasma membrane. TrVLPs are released into the supernatant and used for infection of naïve target cells (**P1**). (**B**) Reporter gene activity in P0 cells. As indicated, NPwt or VP30wt were substituted with the mutants NPΔB56, VP30-A.A_6_.A, or VP30-D.D_6_.D, respectively. Negative controls without L (w/o L) and without VP30 (w/o VP30) were included. P0 cells were harvested for luciferase measurement at 72 hpt. SD is indicated by error bars, and stars indicate statistical significance (*P-value ≤ 0.05, **P-value ≤ 0.01, ***P-value ≤ 0.001, ****P-value ≤ 0.0001). Three independent experiments were performed. (**C**) WB analysis of P0 cell lysates from B using antibodies specific for MARV NP, VP40, or VP30, and for Tubulin. (**D**) Naïve HuH7 cells (**P1**) were infected with concentrated trVLPs from P0. P1 cells were harvested for luciferase measurement 72 hpi; SD is indicated by error bars, and stars indicate statistical significance (*P-value ≤ 0.05; ****P-value ≤ 0.0001). Three independent experiments were performed. (**E**)–(G) show trVLP assays combining VP30 phosphorylation mutants together with NPΔB56, as described in (B)–(D).

To further investigate the role of PP2A on VP30 phosphorylation in the context of the whole viral life cycle, we extended our reporter gene system and established a trVLP assay on the basis of a tetracistronic (4cis) MG that, in addition to the reporter gene, also encodes the structural proteins MARV VP40, GP, and VP24 (Fig. 3A) (22, 40). 4cis trVLP assays represent the most comprehensive life cycle modeling system as the expression of VP40, GP, and VP24 is regulated by genome expression (without overexpression). This assay further recapitulates besides transcription and replication, also transport of assembled nucleocapsids, incorporation into budding particles as well as infectivity and primary transcription in newly infected cells (P1). Similar to 1cis MG assays, producer cells (P0) were transfected with plasmids encoding the viral proteins L, NP, VP35, and VP30 leading to the MG-driven expression of the reporter gene, as well as of the viral proteins VP40, GP, and VP24. As a result, trVLPs are released into the supernatant, which can infect naïve target cells (P1), where primary transcription can be measured based on *Renilla* luciferase activity (22, 40). In line with the results of the 1cis MG assays (Fig. 2C), MARV-specific transcription in producer cells (P0) was comparable in the 4cis trVLP assay setting: while transcription was clearly reduced without VP30 (negative control), or in the NPΔB56 sample, dephosphorylation-mimicking VP30-A.A_6_.A significantly boosted MG transcription (Fig. 3B). Transcription in the presence of the phosphorylation-mimicking VP30-D.D_6_.D was slightly impaired. WB analyses of the P0 cell lysates also confirmed the previously observed phosphorylation-state-dependent shift of VP30, including a hyperphosphorylation of VP30 in the presence of NPΔB56 (Fig. 3C, lane 6). In addition, we stained VP40, which is encoded on the 4cis MG and, therefore, directly reflects MARV-specific transcription efficiency. Expression rates of VP40 correlated with the reporter gene data (Fig. 3C vs. 3B). When using purified trVLPs to infect naive target cells (P1), reporter gene activity reflecting primary transcription was strongly induced in the presence of VP30-A.A_6_.A (Fig. 3D). However, as trVLP formation directly depends on efficient transcription activity in P0 cells, we cannot exclude an overestimation of the effect on primary transcription. VP30-D.D_6_.D led to a slight reduction (Fig. 3D), comparable to producer cells (P0, Fig. 3B). Interestingly, we could not measure reporter gene activity above background in P1 cells in case of trVLPs lacking VP30 (w/o VP30). This suggests an important role for VP30 in early steps in the viral life cycle, as it was previously shown for the generation of recMARV, which was not possible without VP30 (20). The same was true for NPΔB56: although basic reporter gene activity (~50% compared to NPwt) was obtained in P0 cells (Fig. 3B), primary transcription in infected target cells was completely abrogated. These data suggest that the interaction of NP with PP2A-B56 is important for the activation of primary viral transcription in newly infected target cells. We further evaluated whether inefficient VP30 dephosphorylation in the presence of NPΔB56 led to the defect in transcription promotion or if the NP mutant was defective in generating nucleocapsids suitable for RNA synthesis. To examine this, we combined NPΔB56 as well as VP30 phosphomimetic mutants in trVLP assays. Interestingly, decreased reporter gene activity of NPΔB56 could be fully restored in the presence of VP30-A.A_6_.A in the producer cells (Fig. 3E). For the combination of VP30-D.D_6_.D and NPΔB56, a reduction of transcription was measured in trVLP assays similar to NPΔB56 alone (Fig. 3E). WB analyses of the cell lysates revealed again NPΔB56-dependent hyperphosphorylation of VP30wt, while the VP30 phosphomimetic mutants were unaffected (Fig. 3F). After infection of naïve target cells with purified trVLPs, phosphorylation-mimicking VP30-D.D_6_.D was insufficient in supporting primary viral transcription in the presence of NPΔB56. In contrast, VP30-A.A_6_.A could fully rescue the phenotype of NPΔB56 leading to a strong induction of primary viral transcription suggesting general functionality of NPΔB56 in primary viral transcription (Fig. 3G). However, as mentioned above, this effect could be overestimated since trVLP formation directly depends on efficient transcription activity in P0 cells, which was strongly increased in the presence of VP30 A.A_6_.A.

**Fig 4:**
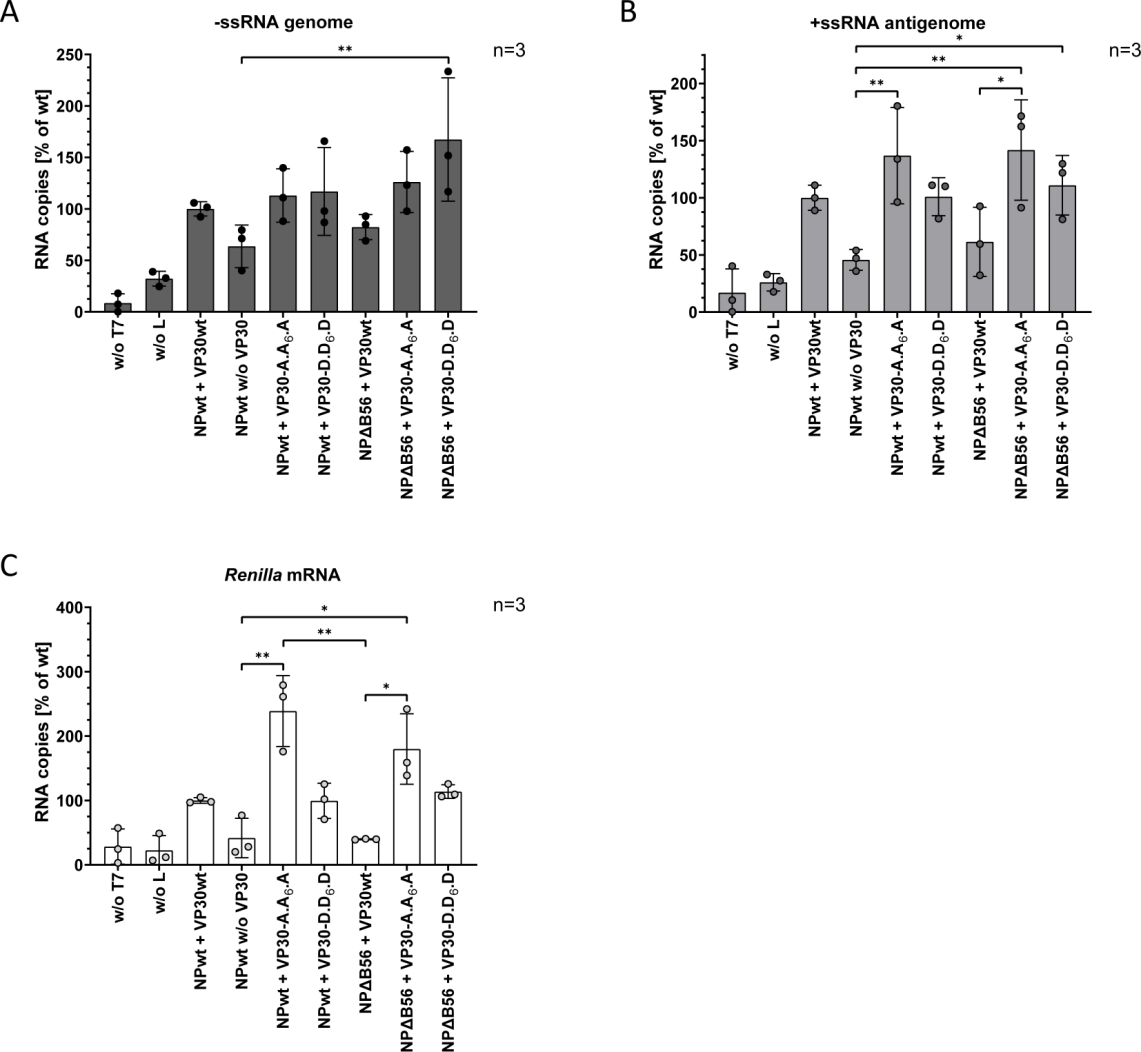
RNA copies of viral RNA species in tetracistronic (4cis) trVLP assays in the presence of VP30 phosphomimetic mutants and NPΔB56. HuH7 cells were transfected with plasmids encoding NPwt, or NPΔB56, and L, VP35, VP30wt, VP30-A.A_6_.A, or VP30-D.D_6_.D, a 4cis MG encoding a *Renilla* luciferase as a reporter gene, as well as a T7 polymerase and a firefly luciferase. Negative controls without T7 polymerase (w/o T7), without L (w/o L), and without VP30 (w/o VP30) were included. Cells were harvested 72 hpt for qRT-PCR analysis using a *Renilla*-specific probe (27). RNA copy values were normalized to positive control (NPwt and VP30wt). SD is indicated by error bars, and stars indicate statistical significance (*P-value ≤ 0.05; **P-value ≤ 0.01). Three independent experiments were performed. (**A**) Genomic RNA (−ssRNA). (**B**) Antigenomic RNA (+ssRNA). (**C**) mRNA of *Renilla* luciferase.

To exclude an effect of the introduced mutations on viral genome replication that would necessarily lead to an increased or decreased primary transcription in infected target cells, we applied a template-specific qRT-PCR approach to differentiate between transcription (mRNA) and replication (−ssRNA) of the MARV-specific MG (27). For this purpose, RNA derived from cell lysates (P0) of 4cis trVLP assays (Fig. 3) was purified. Background levels without L (w/o L) demonstrate T7-driven background of negative-stranded RNA (−ssRNA) synthesis (Fig. 4A) as previously shown (41). Levels of −ssRNA in the absence of VP30 were slightly decreased compared to VP30wt which might suggest a role of MARV VP30 during genome replication. The same was true for genomic RNA synthesis in the presence of NPΔB56. The VP30-A.A_6_.A mutant showed a slight increase in its replication activities compared to the positive control (NPwt + VP30wt), which was even more enhanced in the presence of NPΔB56 (Fig. 4A). Combination of VP30-D.D_6_.D and NPΔB56 further enhanced viral replication, suggesting a positive influence of VP30 phosphorylation on replication, similar to EBOV VP30 (Fig. 4A).

Positive strand antigenomic RNA synthesis (Fig. 4B) as well as mRNA synthesis (Fig. 4C) revealed a picture consistent with the luciferase reporter gene data: dephosphorylation of VP30 (VP30-A.A_6_.A) strongly increased antigenomic (+ssRNA) and mRNA levels, while it was abrogated in the presence of NPΔB56 and hyperphosphorylated VP30 (Fig. 4B and C, NPΔB56 + VP30 wt). Again, VP30-A.A_6_.A rescued the defects in mRNA and +ssRNA synthesis upon missing interaction with PP2A (NPΔB56 + VP30 A.A_6_.A) suggesting that NPΔB56 is functional regarding RNA synthesis. In contrast, antigenomic RNA as well as mRNA synthesis was unaffected in the presence of VP30-D.D_6_.D mutant.

**Fig 5:**
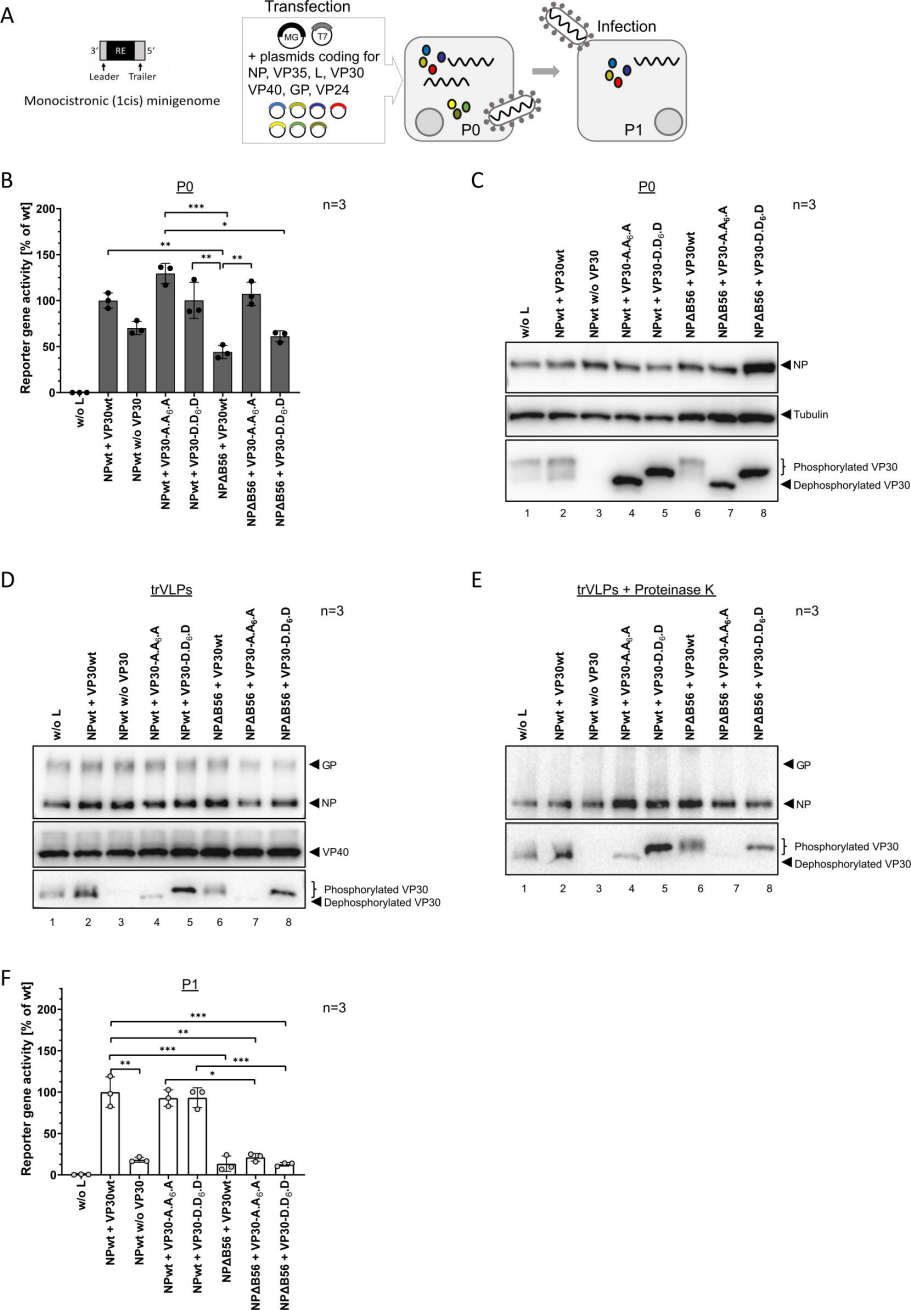
(**A**) Schematic representation of a trVLP assay using a monocistronic (1cis) MARV-specific MG. Cells (**P0**) were transfected with plasmids encoding the viral nucleocapsid proteins NP, L, VP35, and VP30 together with the 1cis MG encoding a *Renilla* luciferase as reporter gene. In contrast to the 4cis MG, the structural viral proteins VP40, GP, and VP24 are expressed in that assay from individual plasmids, allowing the generation of comparable amounts of trVLPs. Additionally, plasmids coding for a T7 polymerase and a firefly luciferase were transfected. After transcription and replication of the 1cis MG, new nucleocapsids assemble and are transported to the plasma membrane. TrVLPs are released into the supernatant, concentrated by ultracentrifugation, and used for infection of naïve target cells (**P1**). (**B**) Reporter gene activity in P0 cells. As indicated, NPwt and/or VP30wt were substituted with the mutants NPΔB56, VP30-A.A_6_.A, or VP30-D.D_6_.D, respectively. Negative controls without L (w/o L) and without VP30 (w/o VP30) were included. P0 cells were harvested for luciferase measurement 72 hpt; SD is indicated by error bars, and stars indicate statistical significance (*P-value ≤ 0.05, **P-value ≤ 0.01, ***P-value ≤ 0.001). Three independent experiments were performed. (**C**) WB analysis of P0 cell lysates from B using antibodies specific for MARV NP, VP40, or VP30, and for tubulin. (**D**) Supernatants from B were ultracentrifuged to concentrate trVLPs for WB analysis using antibodies specific for MARV GP, NP, VP40, and VP30. (**E**) trVLPs from D were additionally treated with proteinase K to digest non-specifically incorporated proteins. (**F**) Generated trVLPs from B were used for infection of naïve HuH7 cells (**P1**). P1 cells were harvested for luciferase measurement 72 hpi; SD is indicated by error bars, and stars indicate statistical significance (*P-value ≤ 0.05, **P-value ≤ 0.01, ***P-value ≤ 0.001). Three independent experiments were performed.

We further investigated whether incorporation of VP30 into trVLPs was affected upon introduction of the respective mutations which could contribute to impaired or increased primary transcription in P1 target cells (Fig. 3D and G). For this purpose, we utilized a trVLP assay that was based on a 1cis MG (Fig. 5A). In contrast to the 4cis trVLP assay, the expression of the structural proteins VP40, GP, and VP24 is driven by individual plasmids in this setup. This approach has the advantage of analyzing the incorporation of viral proteins into comparable amounts of trVLPs, which is independent from efficient MG transcription (Fig. 5A). Reporter gene activity in P0 cells confirmed our previous results obtained with the 4cis MG with impaired transcription efficiency in the presence of NPΔB56 and the boosting effect of dephosphorylation-mimicking VP30 (VP30-A.A_6_.A, Fig. 5B). The expression of viral proteins in P0 cell lysates was controlled by WB analyses (Fig. 5C). Next, the incorporation of NP and VP30 into trVLPs was examined after purifying trVLPs from P0 supernatants via ultracentrifugation and subsequent WB analyses (Fig. 5D and E). VP40 staining confirmed that the overall amount of trVLPs was comparable between samples (Fig. 5D). To confirm specific incorporation, trVLPs were additionally treated with Proteinase K that digests proteins not protected by membranes, such as GP (Fig. 5E). Both NPwt and NPΔB56 were incorporated into trVLPs in similar amounts, indicating that comparable quantities of nucleocapsids were incorporated in the trVLPs, also in the presence of the different phosphomimetic VP30 mutants (Fig. 5D and E, lanes 2–5 vs 6–8). Noteworthy, VP30 incorporation into trVLPs was strongly influenced by its phosphorylation state. While VP30wt was incorporated in both dephosphorylated and phosphorylated form (Fig. 5D and E, lane 2), dephosphorylation-mimicking VP30-A.A_6_.A showed a decreased incorporation in the presence of both NPwt and NPΔB56 (Fig. 5D and E, lanes 4 and 7). In contrast, phosphorylation-mimicking VP30-D.D_6_.D as well as hyperphosphorylated VP30wt in the presence of NPΔB56 was strongly incorporated into trVLPs (Fig. 5D and E lanes 5, 6, and 8). Additionally, we infected naïve target cells (P1) with the generated trVLPs. In line with the data obtained with the 4cis MG trVLPs (Fig. 3D), primary transcription was completely inhibited in case of trVLPs lacking VP30 or in the presence of NPΔB56 (Fig. 5F). TrVLPs with NPwt containing either dephosphorylation- or phosphorylation-mimicking VP30 were able to support primary viral transcription efficiently (Fig. 5F) as seen with the 4cis trVLPs (Fig. 3D). In contrast, 1cis trVLPs containing NPΔB56 were not suitable for primary transcription, neither in the presence of VP30wt, phosphorylation-mimicking VP30-D.D_6_.D, or VP30-A.A_6_.A (Fig. 5F).

Taken together, our data show that the interaction of NP with PP2A-B56 is crucial for the activation of primary transcription in newly infected target cells.

### Influence of the VP30 phosphorylation state on its localization during active RNA synthesis

**Fig 6:**
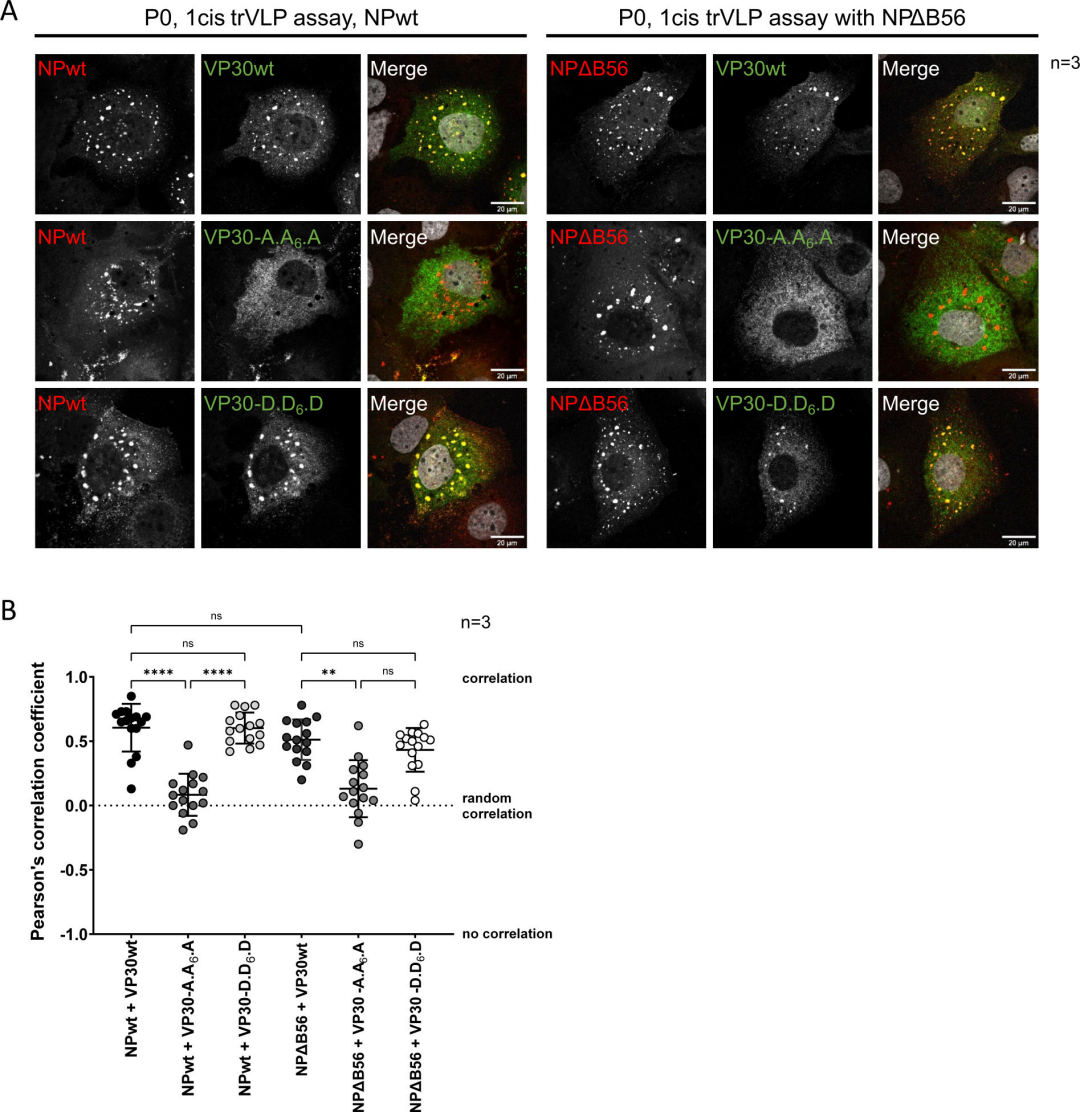
Colocalization of NP and VP30 in P0 of a monocistronic (1cis) trVLP assay. (**A**) HuH7 cells were transfected with plasmids encoding NPwt, or NPΔB56, and L, GP, VP40, VP35, VP24, and VP30wt, VP30-A.A_6_.A, or VP30-D.D_6_.D, a 1cis MG encoding a *Renilla* luciferase as well as a T7 polymerase and a firefly luciferase. Cells were fixed 72 hpt with 4% PFA, and immunofluorescence analysis was performed using NP- (red), and VP30- (green) specific antibodies. Nuclei were stained with DAPI (gray). Scale bar: 20 µM. Three independent experiments were performed. (**B**) Quantification of NP–VP30 colocalization shown in A via PCC. SD is indicated by error bars, and stars indicate statistical significance (ns, not significant, **P-value ≤ 0.01, ****P-value ≤ 0.0001).

As MARV RNA synthesis and nucleocapsid assembly occurs in characteristic inclusion bodies (12, 13, 37), we wanted to localize the phosphomimetic VP30 mutants during active viral RNA synthesis. Previous co-expression data of NP and VP30 have already elucidated that dephosphorylation of VP30 at serine 40 and serine 42 influences its recruitment in NP-induced inclusions due to impaired VP30-NP interaction (24). However, as dephosphorylated VP30 is a strong activator of viral transcription (18), one would expect the recruitment of VP30 to the sites of active viral transcription and replication. To address this question, we performed immunofluorescence analysis of cells that have been transfected with all components of 1cis trVLP assays in the presence of both VP30 phosphomimetic mutants and NPΔB56. During active viral RNA synthesis, we primarily detected enriched VP30wt and VP30-D.D_6_.D in NPwt-induced viral inclusion bodies (Fig. 6A, left panel, upper and lower series). In contrast, VP30-A.A_6_.A colocalized significantly less with NPwt (Fig. 6B), showing a rather diffuse distribution in the cytoplasm (Fig. 6A, left panel, middle series). Compared to NPwt, NPΔB56 did not influence the localization of either VP30wt, VP30-A.A_6_.A, or VP30-D.D_6_.D (Fig. 6A, right panel, Fig. 6B).

In summary, dephosphorylation of VP30 exhibited significantly reduced recruitment to NP-induced viral inclusion bodies as sites of viral RNA synthesis despite its strong activation of viral transcription.

### Dynamic de-/phosphorylation of VP30 by PP2A-B56 is crucial for the rescue of recMARV

**Fig 7:**
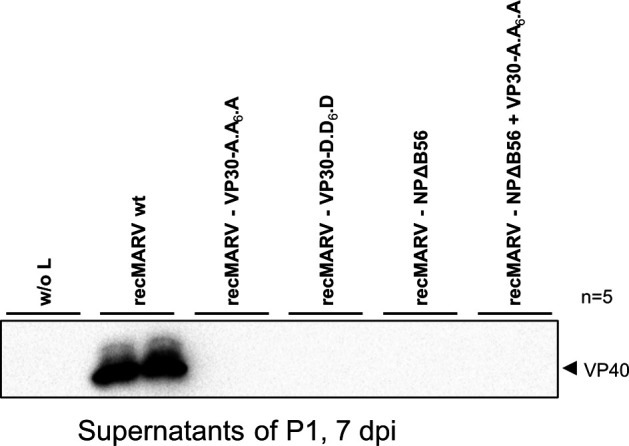
Rescue of recMARV containing NP and/or VP30 mutations. A mixture of Vero E6 and HuH7 cells was transfected with the corresponding plasmids for virus generation (**P0**). Cells were monitored 14 days for CPE formation. At 7 dpt, fresh Vero E6 cells (**P1**) were infected with the supernatants of the P0 cells. Cells were monitored for 14 days for CPE formation. Supernatants of P1 were harvested at day 7 post infection to detect viral proteins via WB analysis. The presence of viral proteins indicated successful MARV rescue. Passaging was continued until passage 3, including WB analysis and CPE detection. WB analysis was performed with a monoclonal antibody detecting MARV VP40. Technical duplicates are shown. Five independent experiments were performed.

After investigating the role of PP2A-driven VP30 dephosphorylation in life cycle modeling systems, we aimed to examine their impact in infection studies by generating recMARV. Therefore, FL plasmids encoding the whole MARV genome, including respective mutations in VP30 (VP30-A.A_6_.A or VP30-D.D_6_.D), or NP (NPΔB56) or a combination of both (VP30-A.A_6_.A + NPΔB56) were cloned to generate recMARV as described elsewhere (30, 33). In P0 cells, we used wt MARV helper plasmids, including NPwt and VP30wt for rescuing the different recMARV to enable sufficient initial transcription and replication of the genomes independently of the introduced mutations. While recMARVwt could successfully be rescued in at least one technical duplicate in each experiment (Fig. 7; Table 1), we were not able to rescue a recMARV with dephosphorylation-mimicking VP30 (VP30-A.A_6_.A) in several attempts (Fig. 7; Table 1) although trVLPs with this mutant infected naïve P1 cells in our trVLP assays (Fig. 3D and 5F). The same also applied to recMARV expressing VP30-D.D_6_.D (Fig. 7; Table 1), which enabled primary viral transcription in both of our trVLP assays as well. These data suggest that a dynamic de- and rephosphorylation of VP30 is essential for the generation of recMARV and, hence, for an efficient viral replication cycle. As could be expected from our previous reporter assay data, we were not able to rescue recMARV carrying NPΔB56 gene, neither in combination with VP30wt, or VP30-A.A_6_.A encoded from the FL genome.

**TABLE 1:** Rescue experiments of different FL MARV mutants

recMARV	Positive rescue duplicate
recMARV wt w/o L (negative control)	0/10
recMARV wt (positive control)	9/10
recMARV-VP30-A.A_6_.A	0/10
recMARV-VP30-D.D_6_.D	0/10
recMARV-NPΔB56	0/10
recMARV-VP30-A.A_6_.A + NPΔB56	0/10

Those results demonstrate that the interaction between NP and PP2A-B56 via the specific binding motif is fundamental for the MARV replication cycle and cannot be compensated by introducing counteracting mutations into VP30 (VP30A.A_6_.A).

### PP2A inhibition reduces MARV replication

**Fig 8:**
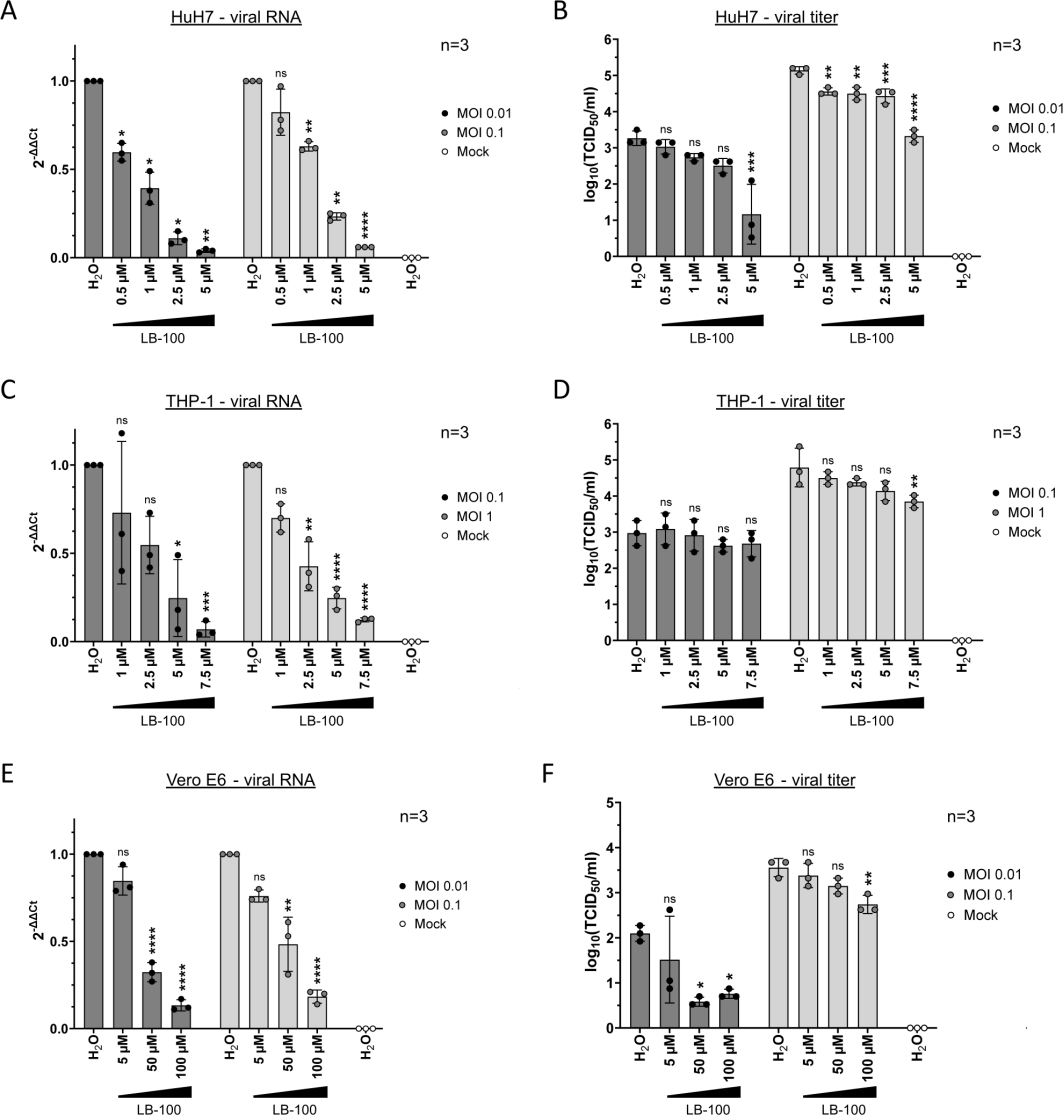
MARV infection in the presence of PP2A inhibitor LB-100. HuH7, THP-1, or Vero E6 cells were infected with MARV (as indicated MOI 0.01, MOI 0.1, or MOI 1) and treated with H_2_O (negative control), or indicated concentrations of LB-100. (**A**, **C**, and** E**) Cells were harvested 24 hpi, RNA was purified and analyzed via qRT-PCRs using MARV VP40 specific primers, as well as 18S specific primers (HuH7 cells and THP-1 cells), or Tubulin-specific primers (Vero E6) for cellular normalization. (**B**, **D**, and **F**) Viral titers. Supernatants from infected cells were harvested for infectious viral titer analyses (TCID_50_/mL). SD is indicated by error bars, and stars indicate statistical significance (ns, not significant, *P-value ≤ 0.05; **P-value ≤ 0.01, ***P-value ≤ 0.001, ****P-value ≤ 0.0001). Three independent experiments were performed.

Lastly, we aimed to assess the role of PP2A during an authentic MARV life cycle by inhibiting its activity. Ectopic expression of the PP2A-B56-specific peptide inhibitor (YFP-LxxIxE) was not suitable in MARV infection models (data not shown). The same was true for YFP-LxxIxE or YFP-AxxAxA-inducible HEK293F cell lines used previously in EBOV infection experiments (27) as these cells did not support MARV replication efficiently (data not shown). Therefore, we assessed the impact of PP2A by inhibiting its phosphatase activity using LB-100 in various cell lines of relevant species: HuH7, Vero E6, and human macrophage-like THP-1, the latter presenting MARV primary target cells (42–44). Dose-dependent cytotoxic effects of LB-100 in HuH7, THP-1, and Vero E6 were determined at 24 hours post treatment using cell viability assays (Fig. S1B through D). Doses of 7.5 µM LB-100 (HuH7) or 10 µM (THP-1) and higher negatively impacted cell viability compared to the water control (Fig. S1B and C) (45). Therefore, we chose LB-100 concentrations between 0.5–5 µM (HuH7) and 1–7.5 µM (THP-1) for MARV infection studies, respectively. In contrast, higher concentrations of LB-100 were applied to Vero E6 cells, which tolerated LB-100 treatment up to 100 µM (Fig. S1D). Cells were infected with MARV at two MOIs as indicated, washed to remove input virus, and then incubated for 24 h in medium containing water (negative control), or various concentrations of LB-100 (as indicated). Afterward, cells were harvested, and MARV RNA copy levels were analyzed via qRT-PCRs (Fig. 8A, C and E). We observed a strong dose-dependent reduction of MARV genomic RNA levels in both human cell lines (HuH7 and THP-1 cells) for both tested MOIs (Fig. 8A and C) as well as for non-human primate-derived cells (Vero E6, Fig. 8E). Concurrently, TCID_50_ analyses were performed to confirm reduction of infectious virus particles in the supernatant of the cells. To confirm MARV replication and exclude analyses of input virus, supernatants from 0 hpi were additionally titrated (data not shown). HuH7 cells exhibited a dose-dependent reduction in released infectious virus particles upon treatment of LB-100 (Fig. 8B). Decreasing MARV titers upon LB-100 treatment were also observed in THP-1 and Vero E6 cells although less pronounced compared to HuH7 cells (Fig. 8D and F). In summary, LB-100 dose-dependently reduced intracellular genomic MARV RNA and infectious virus particles in HuH7, THP-1, and Vero E6 cells, highlighting the conserved role of PP2A for MARV replication independent of cell types and species.

## DISCUSSION

Reversible protein phosphorylation, a critical posttranslational modification, not only regulates cellular signaling pathways but also serves as a key mechanism in viral infections (46). Viruses, such as EBOV and MARV, manipulate cellular kinases and phosphatases to modulate viral protein phosphorylation, impacting viral RNA synthesis and host interactions. Understanding the mechanistic landscape of protein phosphorylations during viral infections unveils potential targets for antiviral interventions. MARV VP30, like EBOV VP30, undergoes phosphorylation that negatively regulates transcription activity, highlighting the role of phosphorylation dynamics in viral replication (18, 23, 24, 47). Our data on the interplay between MARV NP, VP30, and host phosphatase PP2A highlight an essential and conserved mechanism that is exploited by different filoviruses (27), offering potential pan-filovirus therapeutic strategies.

In this study, we confirmed that PP2A directly binds via its regulatory subunit B56 to the conserved binding motif in MARV NP, as already demonstrated for EBOV (27). This interaction is absolutely essential for viral transcription and the generation of recMARV, as indicated by the inability to generate a recMARV lacking PP2A-B56-interaction in several attempts. The absence or inhibition of the interaction between PP2A-B56 and NP resulted in an increased VP30 (hyper)phosphorylation, which subsequently decreased MARV transcription in various life cycle modeling assays. This suggests that dephosphorylation of VP30 occurs at the interface between NP and PP2A-B56 in a trimeric complex. However, we cannot exclude that the hyperphosphorylation itself, that exceeds VP30wt levels, negatively impacts primary viral transcription independently of PP2A-B56. Dephosphorylation-mimicking VP30-A.A_6_.A could restore transcriptional activity in the presence of NPΔB56 or PP2A inhibitors but only in P0 cells and not with respect to primary transcription in an infection scenario (neither in 1cis trVLP assays nor during viral rescue). While this result demonstrates a basic functionality of NPΔB56 during viral RNA synthesis in P0 cells, as also shown by the quantification of different viral RNA species, it further suggests a potential additional inhibitory effect of the introduced mutations during infection that are independent from impaired VP30 dephosphorylation. As NP itself is also a phosphoprotein, one could speculate that PP2A-B56 additionally influences NP’s phosphorylation status, that might impact certain functions of NP, as shown before (47). For EBOV NP, it was recently suggested that interaction with host phosphatase PP1 contributes—in addition to VP30—to the dephosphorylation of NP itself, which affected nucleocapsid formation (48). It can be hypothesized that the dephosphorylation of MARV NP by host phosphatases, such as PP2A, plays a similar role in MARV nucleocapsid formation and transport. Consequently, altered NP (de)phosphorylation may explain the observed impairment in the infectivity of NPΔB56-containing trVLPs.

Although reporter gene activity was enhanced in case of dephosphorylated VP30, even in primary transcription using trVLP assays as demonstrated by our data and Tigabu et al. (18), we were not able to rescue a virus (recMARV-VP30-A.A_6_.A, recMARV-VP30-D.D_6_.D) without having phosphorylatable serine/threonine residues in the VP30 phospho-specific cluster. These data indicate that dynamic cycles of VP30 de- and rephosphorylation are a prerequisite for VP30’s function during the whole viral life cycle, as it has been demonstrated for EBOV VP30 (23, 49, 50).

For EBOV, it is assumed that the dynamic phosphorylation of VP30 facilitates the interaction of VP30 with NP and nucleocapsids to allow its incorporation into budding particles (51, 52). However, in the newly infected cell, induction of viral transcription is driven upon VP30 dephosphorylation (50, 52, 53). In previous studies, the introduction of phosphorylation-mimicking mutations in MARV VP30 did not affect its interaction with NP during recombinant expression, while it was significantly reduced upon VP30 dephosphorylation (18, 24). This was also confirmed here, localization and recruitment of VP30-D.D_6_.D in NP-induced inclusion bodies during transcription and replication processes were unaffected upon introduction of phosphorylation-mimicking aspartic acid residues. In contrast, the dephosphorylation-mimicking VP30-A.A_6_.A was diffusely distributed in the cell similar to previous studies that co-expressed only phosphomimetic VP30 mutants and NP (24). This could also explain the reduced incorporation of VP30-A.A_6_.A into trVLPs (Fig. 5D and E), as NP inclusion bodies are sites of nucleocapsid assembly (37, 54). Since VP30-A.A_6_.A was fully transcriptional active, even in primary transcription (Fig. 3D and 5F), this suggests that only small amounts of VP30 overlapping with NP are sufficient for the induction of viral transcription in our life cycle model systems. The enhanced incorporation of phosphorylation-mimicking VP30-D.D_6_.D, in contrast to the reduced incorporation of dephosphorylation-mimicking VP30-A.A_6_.A, suggests that phosphorylation of MARV VP30 is primarily important for the interaction with newly assembled nucleocapsids to allow VP30 incorporation into virions, as shown for EBOV (51, 52).

Phosphorylation of EBOV VP30 also negatively affects its association with viral RNA and consequently with polymerase co-factor VP35 (49, 55). Therefore, it is suggested that VP30 dephosphorylation contributes to the regulation of viral RNA synthesis, by being part of the transcription initiation complex. Upon phosphorylation and subsequent lack of RNA- and VP35-interaction, EBOV replication by the classical polymerase complex L-VP35 is favored (49, 55). In contrast to EBOV VP30, the interaction of MARV VP30 with VP35 was impaired by both an imitation of permanent phosphorylation (aspartic acid residues) and permanent dephosphorylation (alanine residues) (18). So far it is unclear whether MARV VP30 directly interacts with the viral RNA at all or binds to specific RNA sequences (22), as demonstrated for EBOV VP30 (55, 56). However, it can be assumed that MARV VP30 phosphorylation has a similar negative impact on RNA binding as demonstrated for EBOV VP30 (55) since the RNA-binding motif identified on EBOV VP30 is conserved within different filoviruses including MARV (57). Furthermore, recent data indicate an important regulatory role of MARV VP30 for transcription initiation at internal genes, especially for GP. The authors hypothesize that certain secondary structures in the RNA genome cause dependence on VP30 at this particular transcription start site. However, it is unclear whether this is achieved through a direct interaction of VP30 with the RNA template or through an interaction with the polymerase complex (22).

Inhibiting PP2A, either by affecting its enzymatic activity (using LB-100) or via blocking the binding pocket of the B56 subunit (YFP-LxxIxE), strongly impaired viral transcription, viral growth, and induced VP30 hyperphosphorylation, emphasizing an essential role of PP2A in the MARV replication cycle. As viral growth was notably decreased upon LB-100 treatment across various cell lines of different host species, this highlights the significance of PP2A for MARV replication, independent of the cell type. Our findings, combined with recent research on PP1 for VP30 dephosphorylation, which demonstrated that blocking PP1 activity by a small compound inhibitor likewise reduced viral titers (18), indicate that both phosphatases, PP1 and PP2A, contribute to an efficient MARV life cycle. Upon specific inhibition of PP2A, however, availability of PP1 was not sufficient to counteract VP30 hyperphosphorylation in our studies. Given the conservation of the interaction motifs between PP2A-B56 and VP30 among different filoviral NP including, e.g., Sudan virus, it is plausible to speculate on a conserved PP2A-driven VP30 phosphorylation mechanism observed in both EBOV (27) and MARV (presented data). This molecular mimicry mechanism of NP, recruiting PP2A via the subunit B56 to induce VP30 dephosphorylation, could be targeted by LB-100 or competitive peptide inhibitors specifically targeting the NP-B56 interaction to potentially develop pan-filoviral therapeutics in the future.

**Fig 9:**
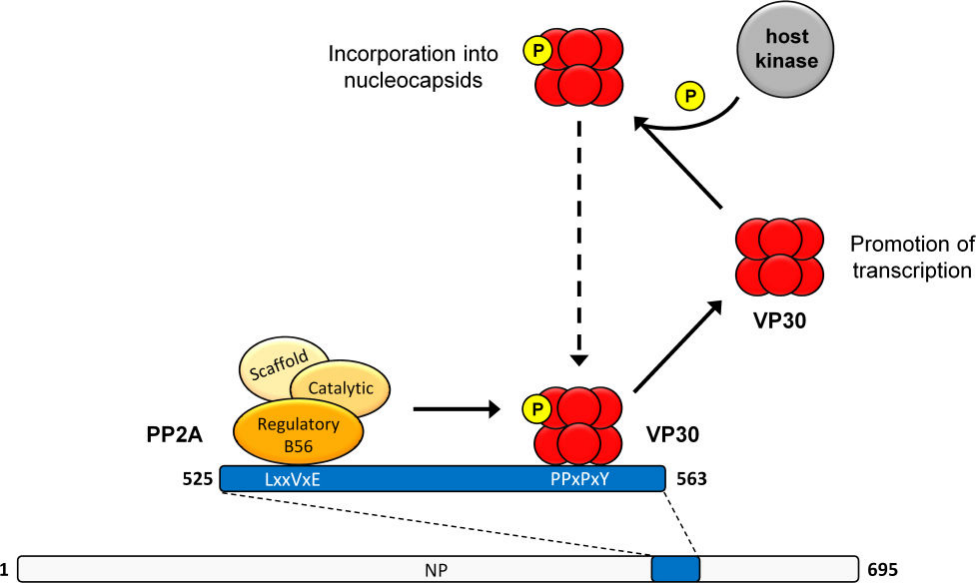
Theoretical model of dynamic VP30 de-/phosphorylation induced by PP2A-B56 and so far unknown host kinase/s. Both VP30 and PP2A-B56 bind to MARV NP as scaffold protein via conserved, specific interaction motifs enabling the dephosphorylation of VP30 by PP2A. In its dephosphorylated state, MARV VP30 promotes viral transcription, whereas phosphorylated VP30 is presumably incorporated in newly assembled nucleocapsids.

In summary, our data demonstrate that host PP2A-B56 plays an essential role during the MARV life cycle (Fig. 9). We hypothesize that MARV NP acts as a scaffold protein, similar to EBOV, bringing the phosphatase PP2A via its subunit B56 and its substrate VP30 into close proximity at the NP interface. This enables VP30 dephosphorylation which is important for efficient MARV transcription. Rephosphorylation of MARV VP30 by so far unknown host kinases could be primarily important for its incorporation into newly assembled nucleocapsids. Consequently, a dynamic change in the phosphorylation status of VP30 is crucial for optimal viral replication.

## Data Availability

The data that support the findings of this study are available from the corresponding author upon request.

## SUPPLEMENTAL MATERIAL

Fig. S1 jvi.01047-24-s0001.tiff Cell viability assays of HEK293F, HuH7, THP-1, and Vero E6 cells treated with inhibitor LB-100.

ASM does not own the copyrights to Supplemental Material that may be linked to, or accessed through, an article. The authors have granted ASM a non-exclusive, world-wide license to publish the Supplemental Material files. Please contact the corresponding author directly for reuse.
