# Supplementary figures and images for: The cellular protein phosphatase 2A is a crucial host factor for Marburg virus transcription
Source: J Virol. 2024 Aug 28;98(9):e01047-24. doi: 10.1128/jvi.01047-24 (PMC11406900; doi:10.1128/jvi.01047-24)

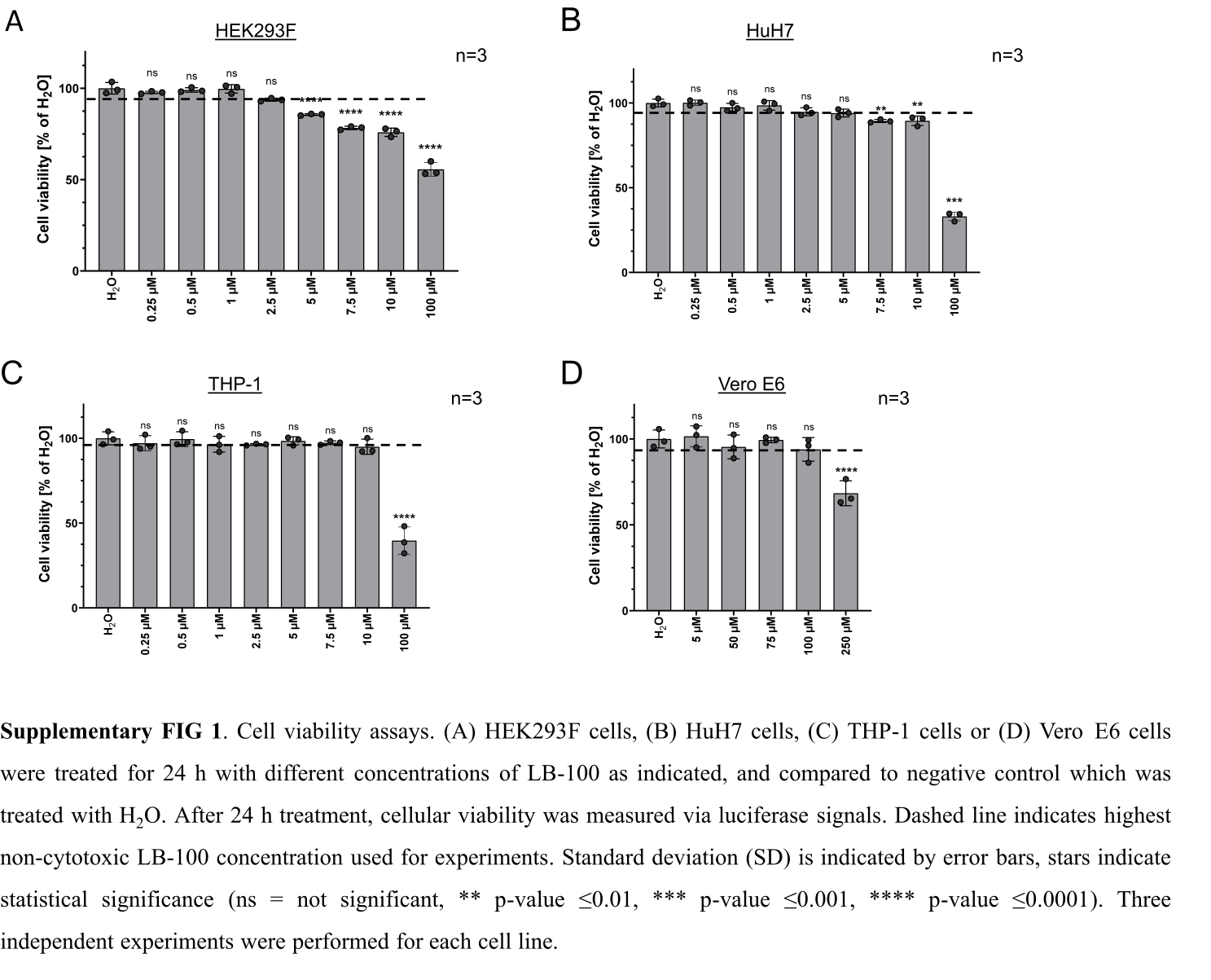

Supplement: Fig. S1 — Cell viability assays of HEK293F, HuH7, THP-1, and Vero E6 cells treated with inhibitor LB-100. [file jvi.01047-24-s0001.tiff]
